# Pharmacological effects of bile acids on polycystic ovary syndrome via the regulation of chemerin

**DOI:** 10.1186/s13020-025-01078-1

**Published:** 2025-04-03

**Authors:** Tian-Tian Tong, Long-Bo Bai, Lee-Fong Yau, Jiu-Yan Li, Hao Huang, Zhi-Hong Jiang

**Affiliations:** 1 State Key Laboratory of Quality Research in Chinese Medicine, Macau Institute for Applied Research in Medicine and Health, Macau University of Science and Technology, Taipa, 999078 Macau SAR China; 2 Jiangxi Province Key Laboratory of Pharmacology of Traditional Chinese Medicine, School of Pharmacy, Gannan Medical University, Ganzhou, 341000 China

**Keywords:** Bile acids, Polycystic Ovarian Syndrome (PCOS), Chemerin isoforms, LC/dynamic MRM-MS, Structure–activity relationships

## Abstract

**Background:**

Polycystic ovary syndrome (PCOS) poses significant health risks for women of reproductive age, and conventional treatments typically involve anti-hormonal interventions or surgical procedures, which often lead to lifelong medication cycles and potential side effects. Bile acids have been applied in the treatment of PCOS-related conditions, including obesity and type 2 diabetes. This study aimed to investigate the effects of bile acids on a PCOS rat model and explore the underlying mechanisms involved.

**Methods:**

Morphological index evaluation, histopathological examination, and hormonal profiling were employed to assess the therapeutic effects of eight bile acids. A targeted proteomics was utilized to characterize and quantify highly homologous chemerin isoforms in rat serum. Network pharmacology analysis was conducted to identify potential targets and molecular mechanisms involved. Molecular docking was performed to evaluate the affinity between bile acids and farnesoid X receptor (FXR).

**Results:**

Five of the eight bile acids markedly restored morphological indices, histopathological manifestations, hormonal imbalances, and chemerin isoform dysregulation. Notably, the therapeutic effects of TDCA and GUDCA on PCOS were reported for the first time. As the severity of the disease decreased, chemerin-157S was negatively correlated with progesterone (P4), estradiol (E2), antral follicles, and corpus luteum, respectively. Several chemerin-associated pathways have been identified via network pharmacology analysis. Additionally, a 7*β*-hydroxy group carried on the steroid skeleton of bile acids has been found to exhibit positive therapeutic efficacy in PCOS.

**Conclusions:**

Downregulating chemerin levels via specific bile acids may be a promising therapeutic strategy for PCOS patients.

**Graphical Abstract:**

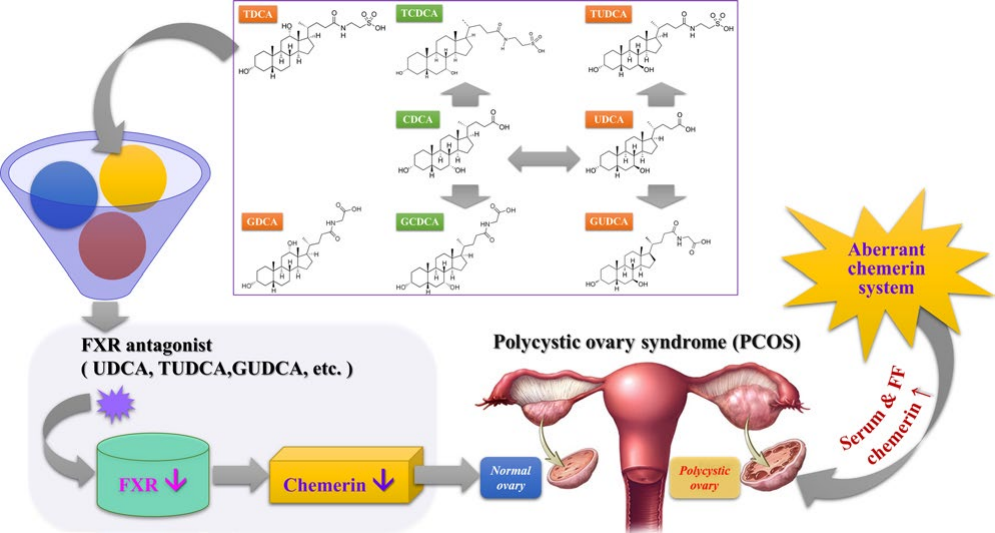

**Supplementary Information:**

The online version contains supplementary material available at 10.1186/s13020-025-01078-1.

## Background

Polycystic ovarian syndrome (PCOS) is a complex endocrine and metabolic disorder commonly observed in women of reproductive age, with a global incidence estimated to be between 6 and 20% [1]. Significantly elevated levels of male sex hormones (hyperandrogenism), ovulatory dysfunction, and enlarged ovaries with fluid-filled sacs (polycystic ovaries) or both are common clinical features of this disease [2]. To prevent serious long-term complications of PCOS, such as type 2 diabetes (T2DM) [3, 4], cardiovascular disease (CVD) [3], metabolic syndrome (glucose intolerance, hypertension, dyslipidemia, and central obesity) [4, 5], and mental health disorders (depression and anxiety disorders) [6], early diagnosis and intervention strategies are urgently needed. Unfortunately, the ambiguous pathogenesis of PCOS makes it difficult to develop ideal medical treatments and early diagnostic markers.

Chemerin, recognized as a multifunctional adipokine, plays a pivotal role in the regulation of immunity, adiposity, and metabolism [7]. Initially, chemerin is secreted as pre-prochemerin, a 163-amino acid-long protein without biological activity. After the N-terminal signal peptide is proteolytically cleaved, it forms prochemerin (chemerin-163S), which is the most common form of circulating chemerin with low biological activity [8, 9]. The carboxy terminus of prochemerin subsequently undergoes proteolytic cleavage by various proteases to produce different chemerin isoforms with diverse biological activities [10]. Multitudinous studies have reported marked upregulation of chemerin expression in granulosa-lutein cells [11], ovarian tissues [12], adipose tissues [13] and serum [14] in PCOS patients compared with normal individuals. Chemerin has been demonstrated to participate in the development of several pathological features of PCOS, including insulin resistance, inflammation, and obesity [15]. While many studies have focused on fluctuations in the levels and biological functions of whole chemerin, emerging studies have begun to delineate the importance of chemerin isoforms in physiological and pathological processes [16–18]. Under normal circumstances, prochemerin is the predominant isoform, with the active isoforms chemerin-157S and chemerin-158 K being less prevalent. However, during inflammation or other pathological states, the levels of chemerin-157S and chemerin-158 K are notably increased [19] due to the proteolytic processing of prochemerin. Thus, the composition and relative abundance of chemerin isoforms vary depending on physiological and pathological conditions. Additionally, the type of predominant chemerin isoform determines whether it has proinflammatory or anti-inflammatory effects [20, 21]. For instance, chemerin-156F has been implicated in the promotion of inflammatory processes and tissue destruction in rheumatoid arthritis (RA) and osteoarthritis (OA) [22]. In stark contrast, chemerin-155A has demonstrated anti-inflammatory effects both in vitro and in a mouse model of leukocyte infiltration. Notably, chemerin-155A has been characterized as a weak antagonist of chemerin-157S in a calcium mobilization assay, suggesting that it can partially neutralize the proinflammatory actions of chemerin-157S by competing for the same receptor [23]. In health and disease, the dynamic interplay among these isoforms may lead to a modulation of total chemerin activity, underscoring the importance of considering isoform-specific contributions when assessing the role of chemerin in biological processes. Therefore, characterizing the specific isoforms of chemerin associated with PCOS and exploring their potential use as biomarkers for disease diagnosis and treatment are essential.

Bear bile has been used in Traditional Chinese Medicine (TCM) clinical practice for thousands of years [24]. Modern chemical research has indicated that bile acids, including ursodeoxycholic acid (UDCA, 0.4%–2.0%), chenodeoxycholic acid (CDCA, 1.0%–5.0%), taurochenodeoxycholic acid (TCDCA, 30.0%–42.0%), tauroursodeoxycholic acid (TUDCA, 38.0%–50.0%), and taurocholic acid (TCA, 0.5%–2.5%), are the major components of bear bile powder [25]. Bile acids have been extensively applied in the treatment of PCOS-related conditions including obesity, T2DM, and non-alcoholic fatty liver disease (NAFLD) [26–28]. Furthermore, glycodeoxycholic acid (GDCA) and TUDCA have been verified to have therapeutic effects on insulin resistance, ovarian dysfunction and infertility in PCOS mice [29]. UDCA has been shown to ameliorate ovarian morphology, hormonal imbalances, and insulin resistance in PCOS rats [30]. Despite the structural differences among the three bile acids, including variations in the number and position of the hydroxyl groups, the conjugation with amino acids such as taurine or glycine, and the configuration of the steroid nucleus, they all have therapeutic effects on PCOS. Therefore, it is necessary to conduct a comparative analysis of a series of bile acids to reveal the structure-activity relationship, thereby providing a theoretical basis for the design and optimization of bile acid drugs for the treatment of PCOS.

In our previous study [31], we reported that specific chemerin isoforms were significantly elevated in PCOS patients compared with healthy individuals, suggesting their potential to serve as biomarkers for this disease. The present study was designed to confirm this finding by quantifying the levels of specific chemerin isoforms in a PCOS rat model group compared with a control group. Moreover, we aimed to investigate the effects of bile acids on a PCOS rat model and explore the underlying mechanisms involved.

## Methods

### Solvent, drugs, and ELISA kits

LC–MS grade acetonitrile (ACN) was purchased from J.T. Baker (Avantor Performance Materials, LLC, Center Valley, PA, USA). LC–MS grade formic acid (HCOOH) and ammonium bicarbonate (ABC) were obtained from Sigma-Aldrich (St. Louis, MO, USA). Mass Spectrometry Grade Trypsin Gold (EC No. 3.4.21.4) was acquired from Promega (Madison, WI, USA). AminoLink® Plus Coupling Resin (Cat No. 20501), Pierce™ Spin Columns-Snap Cap (Cat No. 69725), and other necessary materials were obtained from Thermo Fisher Scientific (Rockford, IL, USA). The TIG2 antibody (E-7, Cat. No. sc-373797) was procured from Santa Cruz Biotechnology (Santa Cruz, CA). Letrozole (LETZ) and clomiphene citrate (CC, Positive control drug) were obtained from Kindin (Hong Kong) Industrial Co., Limited. The following bile acids were purchased from Shanghai Yuanye Bio-Technology Co., Ltd: glycodeoxycholic acid monohydrate (GDCA, CAS: 360-65-6), taurodeoxycholic acid sodium salt (TDCA, CAS: 1180-95-6), chenodeoxycholic acid (CDCA, CAS: 474-25-9), ursodeoxycholic acid (UDCA, CAS: 128-13-2), sodium glycochenodeoxycholate (GCDCA, CAS: 16564-43-5), glycoursodeoxycholic acid (GUDCA, CAS: 64480-66-6), sodium taurochenodeoxycholate (TCDCA, CAS: 6009-98-9), and tauroursodeoxycholic acid (TUDCA, CAS: 14605-22-2). ELISA kits for the following hormones were purchased from Cloud-Clone Corp. (CCC, Katy, TX, USA): testosterone (T, Cat. No. CEA458Ge-96T), estradiol (E2, Cat. No. CEA461Ge-96T), luteinizing hormone (LH, Cat. No. CEA441Ra-96T), follicle stimulating hormone (FSH, Cat. No. CEA830Ra-96T), and progesterone (P4, Cat. No. CEA459Ge-96T).

### Animals

One hundred twenty-three female Sprague–Dawley (SD) rats (average weight, 180 ± 20 g; aged 6–7 weeks; certificate no. 44822700008568) were purchased from the Institute of Analysis, Guangdong Academy of Sciences (China National Analytical Center, Guangzhou, NACC) with the licence of SYXK (Guangdong) 2019-0201. During the study, all the animals were caged in standard polypropylene cages and maintained in a controlled environment at 24–26 °C with 45–65% humidity and a 12 h light/dark cycle. The rats were provided with standard chow and water ad libitum. All procedures were approved by the Animal Care Committee on the Ethics of Animal Experiments of the Institute of Analysis, Guangdong Academy of Sciences (NACC) for the care and use of animals.

### Experimental PCOS rat model and treatment

**Fig. 1:**
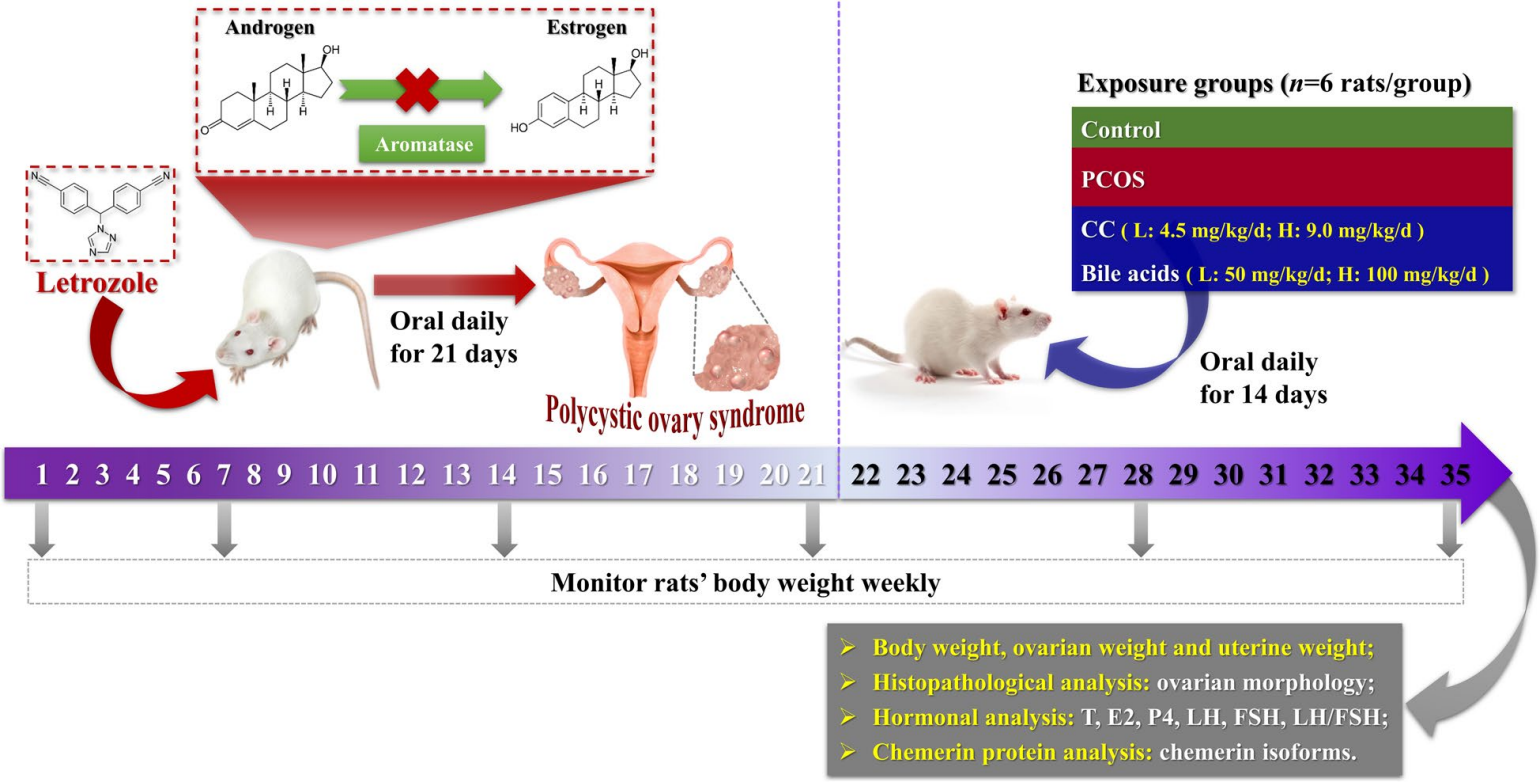
A schematic representation of the animal experiment. This schematic diagram illustrates the experimental timeline and treatment groups for investigating the effects of various bile acids on PCOS rats. The study lasted 35 days and was divided into two phases: an initial 21-day modelling phase with letrozole to induce PCOS, followed by a 14-day treatment phase with different therapeutic agents. This study assesses several endpoints, including body weight, ovarian and uterine weights, histopathological analysis of ovarian morphology, hormonal profiles (testosterone, estradiol, progesterone, luteinizing hormone, follicle-stimulating hormone, and their ratios), and chemerin protein isoforms

After 1 week of acclimatization, the rats were randomly divided into 20 experimental groups (6 animals per group): **Group 1** (Control): six rats received daily oral doses of 4 mL/kg vehicle only (0.5% sodium carboxymethyl cellulose, CMC-Na) for 21 days; the other rats were administered 1 mg/kg letrozole dissolved in 0.5% CMC-Na solution once daily for 21 days to induce PCOS [32] and were further divided into another 19 groups. The rats in **group 1** (Control) and **group 2** (PCOS) were administered 4 mL/kg of vehicle only from day 22 until the end of the experiment; the rats in **group 3** (CC-Low) and **group 4** (CC-High) were the positive control group and were administered 4.5 mg/kg and 9.0 mg/kg CC, respectively; **group 5** (GDCA-Low) and **group 6** (GDCA-High) rats were treated with 50 mg/kg and 100 mg/kg GDCA, respectively; **group 7** (UDCA-Low) and **group 8** (UDCA-High) rats were treated with 50 mg/kg and 100 mg/kg UDCA, respectively; **group 9** (TUDCA-Low) and **group 10** (TUDCA-High) rats were treated with 50 mg/kg and 100 mg/kg TUDCA, respectively; **group 11** (TDCA-Low) and **group 12** (TDCA-High) rats were treated with 50 mg/kg and 100 mg/kg TDCA, respectively; and **group 13** (GUDCA-Low) and **group 14** (GUDCA-High) rats were treated with 50 mg/kg and 100 mg/kg GUDCA; **group 15** (CDCA-Low) and **group 16** (CDCA-High) rats were respectively treated with 50 mg/kg and 100 mg/kg CDCA; **group 17** (GCDCA-Low) and **group 18** (GCDCA-High) rats were respectively treated with 50 mg/kg and 100 mg/kg GCDCA; **group 19** (TCDCA-Low) and **group 20** (TCDCA-High) rats were respectively treated with 50 mg/kg and 100 mg/kg TCDCA. All the groups were orally administered their respective treatments for 14 days (from days 22 to 35). All the rats were weighed every week to observe the changes in weight and calculate drug doses. A detailed schematic of the animal treatment is shown in Fig. 1.

### Collection of tissue and blood samples

Following the intervention, the rats were subjected to a 12-h fast with free access to water. The rats were subsequently anaesthetized with 2% pentobarbital sodium at a dosage of 100 μg/g of body weight. Peripheral blood samples were then collected from the abdominal aorta. Two sets of duplicate samples were drawn: one set was placed in sodium heparin tubes for plasma separation, and the other set was placed in separation tubes without anticoagulant for serum separation. Both the serum and plasma samples were separated via centrifugation at 4 °C at 3000 rpm for 10 min. The supernatant was then carefully transferred to a new sterile microcentrifuge tube and stored at − 80 °C until further analysis. After the rats were sacrificed, the uteri and ovaries were excised, weighed, and analysed to evaluate the effects of the test compounds on the reproductive organs. The ovaries were fixed in a 4% paraformaldehyde solution for 24 h before being embedded in paraffin for histological examination.

### Histopathological analysis

Ovaries were fixed in a 4% paraformaldehyde solution, routinely processed, and embedded in paraffin. The paraffin-embedded ovarian tissues were sectioned via a microtome, and four-millimeter-thick sections were cut onto glass slides and stained with hematoxylin–eosin (H&E) for microscopic examination (magnification, ×40 and ×200). Transverse sections of the left and right ovaries of all the rats were prepared and subjected to histological evaluation. The ovarian sections were assessed for the presence and number of corpus luteum, antral, atretic, and cystic follicles. The corpus luteum was typically very large relative to the size of the ovary, showing a convoluted layer of mainly granulosa lutein cells surrounding a fibrous to hemorrhagic center. Theca and granulosa lutein cell layers were clearly distinguishable. Follicles were categorized and counted on the basis of the following definitions [33–35]. Antral follicles were characterized by the presence of a nucleated oocyte and at least two layers of cuboidal granulosa cells, irrespective of the prominence of the lumen. Atretic follicles were identified by the presence of a degenerated zona pellucida or oocytes, pyknotic granulosa cells, and cellular debris within the antral cavity. Cystic follicles were recognized as large, fluid-filled structures with a thinned granulosa cell layer and a thickened theca interna cell layer.

### Hormonal analysis

The plasma levels of T, E2, LH, FSH and P4 were determined via enzyme-linked immunosorbent assay (ELISA) kits following the manufacturers’ protocols. Measurements were performed with a SpectraMax 190 microplate reader (Molecular Devices, Sunnyvale, USA). The analytical sensitivities of each ELISA kit were as follows: 152.50 pg/mL for T, 4.45 pg/mL for E2, 37.59 pg/mL for LH, 1.11 ng/mL for FSH and 0.47 ng/mL for P4. The standard curves for the assays spanned the following ranges: 370.40–30,000.00 pg/mL for T, 12.35–1000.00 pg/mL for E2, 98.77–10,000.00 pg/mL for LH, 2.47–200.00 ng/mL for FSH and 1.23–100.00 ng/mL for P4. Hormone concentrations were determined via calibration curves established from the calibrators supplied with the kits. For each sample, quantification was performed on three replicates.

### Analysis of various chemerin isoforms

A well-established liquid chromatography/dynamic multiple reaction monitoring-mass spectrometry (LC/dynamic MRM-MS)-based targeted proteomic approach was used in our laboratory for the characterization and quantification of highly homologous chemerin isoforms in human biofluids from PCOS patients and healthy donors, as previously described [31]. Here, the concentrations of different chemerin isoforms in rat serum from the PCOS group, control group, and different treatment groups were determined via the abovementioned methods with slight modifications, and each sample was technically repeated three times. Briefly, 200 µL of the diluted anti-chemerin antibody (TIG2 antibody) solution (containing 10 µg of antibody) was added to 100 µL of AminoLink Plus Coupling Resin (200 µL of resin slurry), and the reaction mixture was mixed by gentle end-over-end rocking for 4 h at ambient temperature. The resin was subsequently washed with 300 µL of pH 7.2 coupling buffer (0.1 M sodium phosphate, 0.15 M NaCl). In a fume hood, 100 µL of pH 7.2 coupling buffer and 5 M sodium cyanoborohydride solution (2 µL) were added, and the reaction mixture was mixed by gentle end-over-end rocking overnight at 4 °C. Any remaining active sites on the resin were blocked to prevent interactions with analytes during the immunoaffinity purification. The prepared affinity column was subsequently washed with 300 µL of binding buffer (PBS, Thermo Scientific Product No. 28372). Rat serum (1:1 diluted with binding buffer) was added, and the reaction mixture was mixed by gentle end-over-end rocking for 1 h at room temperature. The solution was then removed from the column, and the resin was washed with 300 µL of binding buffer. Thereafter, the bound protein was eluted by applying 800 µL of elution buffer (0.1 M glycine buffer, pH 2.7), and the eluted fractions were collected. The pH of the fractions was adjusted to neutral by adding 40 μL of neutralization buffer (1 M Tris, pH 9.0) per 800 µL of collected eluate. The obtained protein solutions were then transferred to 10 K centrifuge filter units for buffer exchange, and the resulting aqueous solutions were concentrated. The protein samples were subsequently digested with trypsin by dissolving the protein in 50 mM ammonium bicarbonate and exposing the protein to protease (at an enzyme/substrate ratio of 1:50) for 16 h at 37 °C. Thereafter, the tryptic digestion was quenched by the addition of an acidified SIS peptide mixture to obtain a final formic acid concentration of 0.5% v/v with a pH less than 3. The mixture was concentration balanced so that the levels of the SIS peptides approximated the endogenous concentrations of the target proteins. The samples were then centrifuged at 14,000×*g* for 20 min (4 °C). The sample supernatant was collected and subjected to LC/dynamic MRM-MS (an Agilent 6490 Triple Quadrupole mass spectrometer equipped with a 1290 Infinity Quaternary liquid chromatography system) analysis.

### Construction of bile acids targets and the PCOS targets database

The PubChem database (https://pubchem.ncbi.nlm.nih.gov/) was utilized to search for bile acids and retrieve their corresponding Simplified Molecular Input Line Entry System (SMILES) strings. The SMILES strings of eight bile acids were subsequently imported into the SwissTargetPrediction database (http://swisstargetprediction.ch/). This platform was employed to predict the potential targets of these bile acids. The search results were meticulously organized to construct a comprehensive bile acids-target database, facilitating further investigation of the interactions between these compounds and their respective biological targets. A systematic search was conducted in the GeneCards database (https://www.genecards.org/) via the keyword “polycystic ovary syndrome”. The search results were aggregated, and duplicate genes were meticulously removed to form a reliable and non-redundant PCOS disease target database.

### Construction of the protein–protein interaction (PPI) network

To identify the intersection between bile acid targets and PCOS targets, the target points of bile acids were mapped against the disease targets of PCOS. These bile acid targets and PCOS disease targets were then imported into the Venny 2.1.0 online diagramming tool (https://bioinfogp.cnb.csic.es/tools/venny/) to generate a Venn diagram illustrating the targets related to PCOS treatment with bile acids. Subsequently, the related targets for PCOS treatment with bile acids were imported into the STRING database (https://string-db.org/), with the protein species set to “*Homo sapiens*” and the target association confidence threshold set to “high confidence (0.700)”. The isolated nodes were concealed, and the Tab-Separated Values (TSV) data of the target interaction network relationships were downloaded. These data were then imported into Cytoscape 3.9.1 software to construct the PPI network, facilitating data visualization. The maximal clique centrality (MCC) algorithm of the CytoHubba plugin was utilized to screen out important hub targets within the network, facilitating the identification of key proteins involved in the interaction network related to PCOS treatment with bile acids.

### Kyoto encyclopedia of genes and genomes (KEGG) signalling pathway enrichment analysis

The DAVID database (https://davidbioinformatics.nih.gov/) was utilized to conduct KEGG signalling pathway enrichment analysis on the potential targets of bile acids in the treatment of PCOS. This analysis was employed to explore the potential signalling pathways through which bile acids exert their therapeutic effects in the treatment of PCOS. The KEGG pathway enrichment data were subsequently downloaded, and advanced data visualization analyses were conducted.

### Construction of the bile acids-disease targets-signalling pathways network

The targets of bile acids were systematically matched with the targets associated with PCOS. Only those bile acids and disease targets that yielded positive matching results were retained, ensuring the relevance and specificity of the dataset. Data lacking matching results were excluded to maintain the integrity and focus of the analysis. By integrating the enriched key signalling pathways, a comprehensive bile acids-disease targets-signal pathways database was successfully established. To visualize and predict the network relationships among the components, disease targets, and signalling pathways of bile acids in treating PCOS, the database information was imported into Cytoscape 3.9.1 software. The Network Analyser plugin, an integral feature of Cytoscape, was employed to conduct a thorough analysis of the network’s topological parameters. This analysis allowed for the identification of key structural and functional characteristics of the network, such as node connectivity and network density. The core components and core targets involved in the treatment of PCOS were meticulously screened on the basis of the degree of connectivity. This step was crucial in pinpointing the most influential elements within the network, thereby highlighting the potential therapeutic targets, and signalling pathways that are central to the action of bile acids in mitigating the symptoms and progression of PCOS.

### Molecular docking simulation

Molecular docking was conducted to further elucidate the interactions between bile acids and target proteins in the treatment of PCOS. The 3D crystal structures of the bile acids and target proteins were retrieved from the RCSB PDB database (https://www.rcsb.org/), and the corresponding PDB files were downloaded for further analysis. The target protein was subjected to a pre-processing step via the PyMol software, which included the removal of solvent molecules, the addition of hydrogen atoms, and the balancing of charges to prepare the proteins for molecular docking simulations. The 3D structures of bile acids were sourced from the ZINC database (https://zinc.docking.org/) and saved in Mol2 format, which is compatible with molecular docking software. The structural data of both the target protein and bile acids were imported into the AutoDockTools-1.5.6 database for comprehensive molecular docking evaluation analysis. Finally, the docking results were visualized via PyMol software, allowing for a detailed examination of the binding sites and interaction patterns.

### Statistical analysis

Statistical significance was calculated via GraphPad Prism 6 (GraphPad Software, La Jolla, CA, USA). Comparisons were performed with the Mann‒Whitney test for nonparametric continuous variables and Fisher’s exact test for categorical variables. Continuous variables were presented as the mean ± standard deviation (S.D.). A two-sided P value < 0.05 was considered to indicate statistical significance. The sample size was not predetermined by a statistical method. No data points were excluded from the analysis. Initially, we compared the control group with the PCOS group to confirm that the markers in the PCOS group were significantly different. We subsequently compared the study parameters between the treated groups and the PCOS group to assess the effects of the intervention.

## Results

### Effects of eight bile acids on body weight, ovarian weight, and uterine weight

**Fig. 2:**
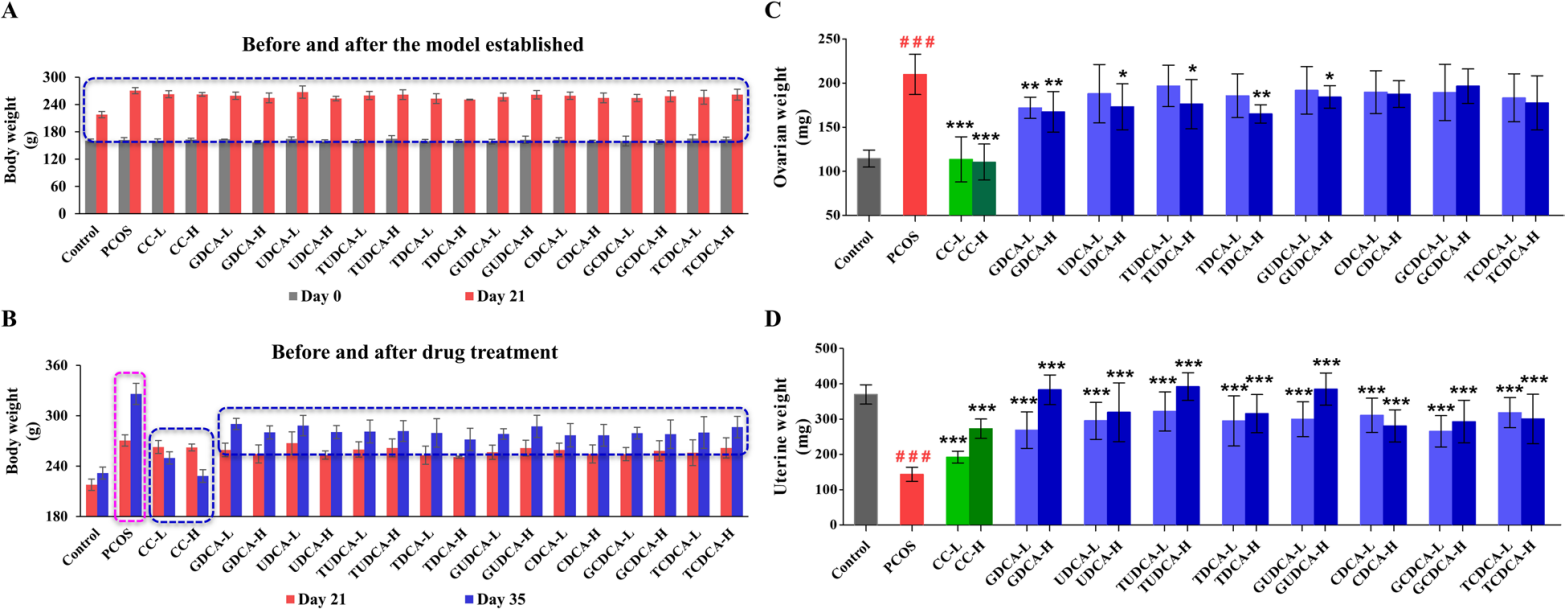
Effects of different bile acid treatments on body, ovarian and uterine weights in a rat model of PCOS (*n* = 6 rats/group). **A** Shows body weights before (Day 0) and after (Day 21) the establishment of the PCOS model. The dashed blue box highlights specific groups for comparison. **B** Illustrates body weights before (Day 21) and after (Day 35) drug treatment. The dashed blue boxe indicates groups with significant weight changes following treatment, and the dashed pink box indicates the weight change of the untreated PCOS group during this period. **C** Displays ovarian weights across different groups, with statistical significance markers indicating differences between groups. **D** Shows uterine weights across the same groups, also with statistical significance markers highlighting differences. All data are presented as the mean ± standard deviation (SD), and statistical significance is denoted by asterisks (*) and hashes (^#^). Hashes (^#^): Indicate significant differences between the PCOS group and the control group (^#^, *P* < 0.05; ^##^, *P* < 0.01; ^###^, *P* < 0.001); Asterisks (*): Indicate significant differences between treatment groups and the PCOS group (*, *P* < 0.05; **,* P* < 0.01; ***, *P* < 0.001)

The body weights of the rats were monitored weekly from the beginning until sacrifice (Fig. 1). The initial body weights of the rats in all the groups were approximately equal at 161.16 ± 2.55 g. After 21 days of LETZ administration, the body weights of the rats significantly increased compared with those of the controls (*P* < 0.001) (Fig. 2A, Supplementary Table 1). At the end of the experiment, all the treated rats (**group 3–20**) presented significantly lower body weights than the untreated PCOS rats (**group 2**, *P* < 0.001) (Fig. 2B, Supplementary Table 1). A significant increase in ovarian weight (*P* < 0.001) and a corresponding decrease in uterine weight (*P* < 0.001) were observed in the rats of **group 2** compared with those of **group 1** (Fig. 2C and D, Supplementary Table 1). Compared with the **group 2**, CC (**group 3** and** 4**) mitigated LETZ-induced effects by reducing ovarian weight (*P* < 0.001) and increasing uterine weight (*P* < 0.001) (Fig. 2C and D, Supplementary Table 1). Concurrently, treatment with GDCA (**group 5** and** 6**, *P* < 0.01), UDCA (**group 8**, *P* < 0.05), TUDCA (**group 10**, *P* < 0.05), TDCA (**group 12**, *P* < 0.01), and GUDCA (**group 14**, *P* < 0.05) significantly decreased ovarian weight compared with that of **group 2** (Fig. 2C, Supplementary Table 1). Compared with **group 2**, the ovarian weights of the other treatment groups did not significantly differ (Fig. 2C, Supplementary Table 1). Additionally, compared with group 2, the uterine weights of the rats in all the treated groups were significantly greater (*P* < 0.001) (Fig. 2D, Supplementary Table 1). In brief, the observed changes in the three weight-related PCOS indicators confirmed the successful establishment of the PCOS rat model. Furthermore, the GDCA, UDCA, TUDCA, TDCA and GUDCA groups presented significant improvements compared with the **group 2**.

### Effects of eight bile acids on rat ovarian morphology

**Fig. 3:**
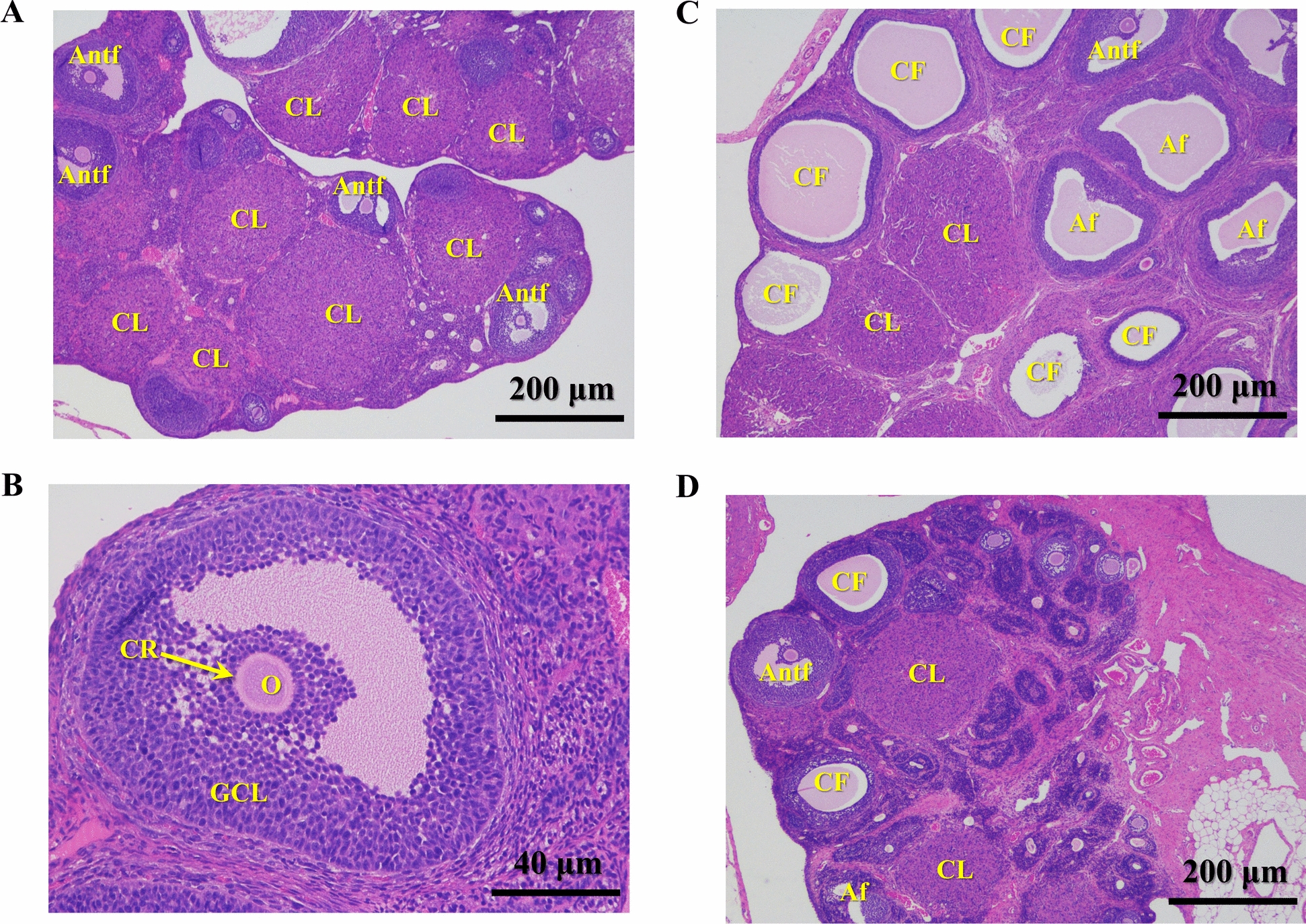
Morphological changes in rat ovarian tissues from the control, PCOS, and CC groups. This set of images presents histopathological sections of ovarian tissue, stained with hematoxylin–eosin (H&E). **A** At 40× magnification, images of ovarian tissue from the control group show multiple corpora lutea (CL) and antral follicles (Antf). The scale bar represents 200 μm. **B** At 200× magnification, image of an antral follicle structure in the control group, illustrating the oocyte (O), corona radiate (CR) and granulosa cell layers (GCLs). The scale bar represents 40 μm. **C** At 40× magnification view of ovarian tissue from the PCOS group, which exhibited numerous cystic follicles (CFs), atretic follicles (Afs), CLs and Antfs. The scale bar represents 200 μm. **D** At 40× magnification, images of ovarian tissue from the positive control group, showing the CL, Antf and Af. The scale bar represents 200 μm

Light microscopy was applied to observe changes in ovarian morphology across all the experimental groups. The rats in the control group presented normal ovarian morphology and colouration, with a notable presence of corpus luteum and antral follicles (Fig. 3A, Supplementary Table 2). Figure 3B magnified the antral follicle region in the ovary of the normal control group rat (Fig. 3A) to 200x (scale bar 40 μm) to more clearly observe multiple layers of granulosa cells, oocytes, and corona radiata within the follicles (Fig. 3B). In contrast, the ovaries of LETZ-induced PCOS rats (**group 2**) presented pathological alterations characterized by the appearance of multiple dilated cystic follicles on the ovarian surface, a significant increase in the number of atresia follicles (*P* < 0.001), and a marked reduction in the number of antral follicles and corpus luteum in the development stage (*P* < 0.001) (Fig. 3C, Supplementary Table 2). The PCOS rats treated with positive control drugs (**group 3** and **4**) almost recovered to normal ovarian morphology (Fig. 3D).

**Fig. 4:**
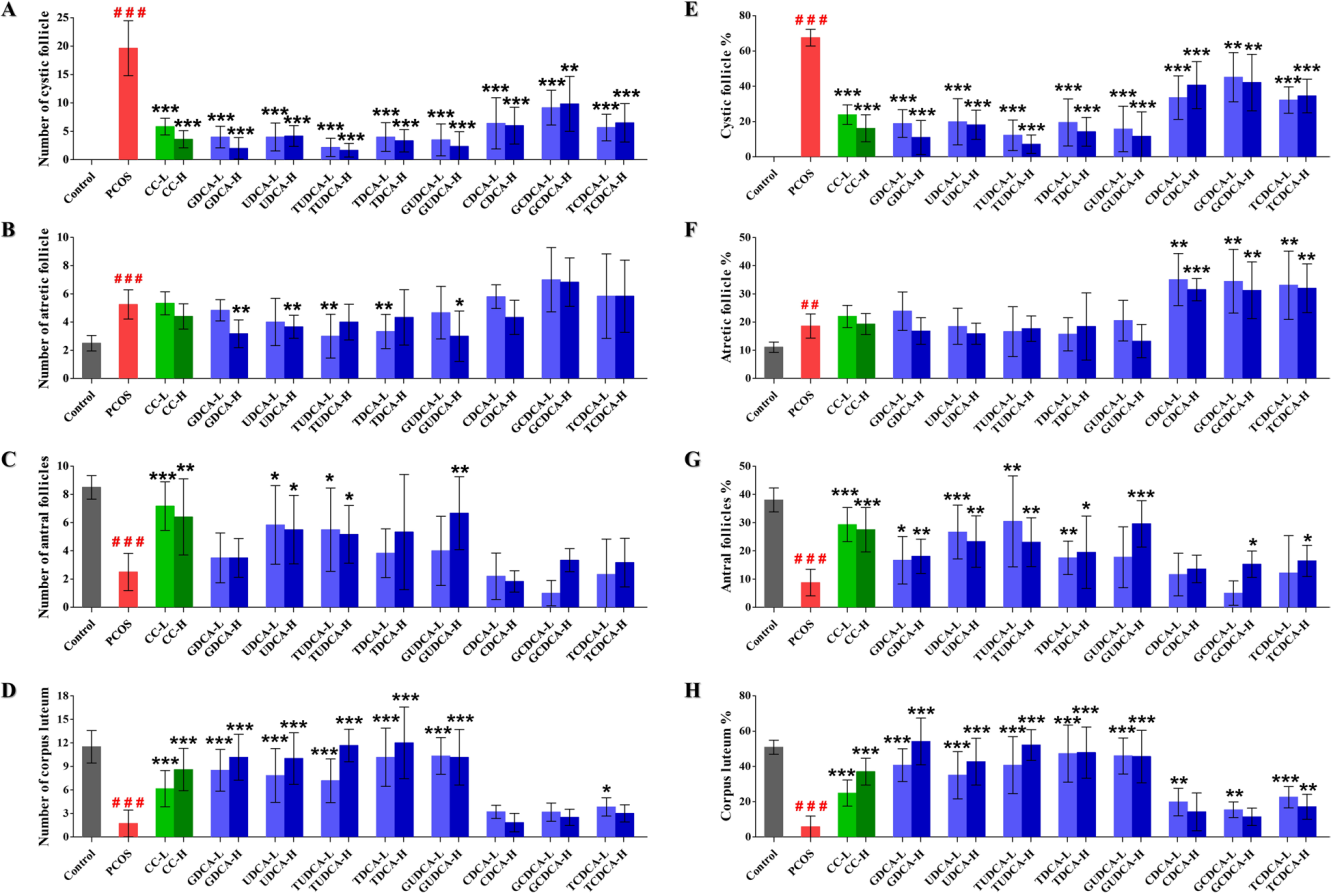
Effects of bile acid treatments on ovarian histopathological parameters in PCOS. This figure presents the impact of various bile acid treatments on different ovarian histopathological parameters in a PCOS model. The eight subfigures (**A**–**H**) depict measurements across different groups, including control, PCOS, and multiple treatment groups with low and high doses of different bile acids. **A** Number of cystic follicles: Shows the count of cystic follicles across groups; **B** Number of atretic follicles: Illustrates the count of atretic follicles; **C** Number of antral follicles: Displays the count of antral follicles; **D** Number of corpus luteum: Shows the count of corpus luteum across groups; **E** Cystic follicle percentage: Displays the percentage of cystic follicles in all parameters; **F** Atretic follicle percentage: Illustrates the percentage of atretic follicles in all parameters; **G** Antral follicle percentage: Displays the percentage of antral follicles in all parameters; **H** Corpus luteum percentage: Shows the percentage of corpus luteum in all parameters. All data are presented as the mean ± standard deviation (SD), and statistical significance is denoted by asterisks (*) and hashes (^#^). Hashes (^#^): Indicate significant differences between the PCOS group and the control group (^#^, *P* < 0.05; ^##^, *P* < 0.01; ^###^, *P* < 0.001); Asterisks (*): Indicate significant differences between treatment groups and the PCOS group (*, *P* < 0.05; **,* P* < 0.01; ***, *P* < 0.001)

**Fig. 5:**
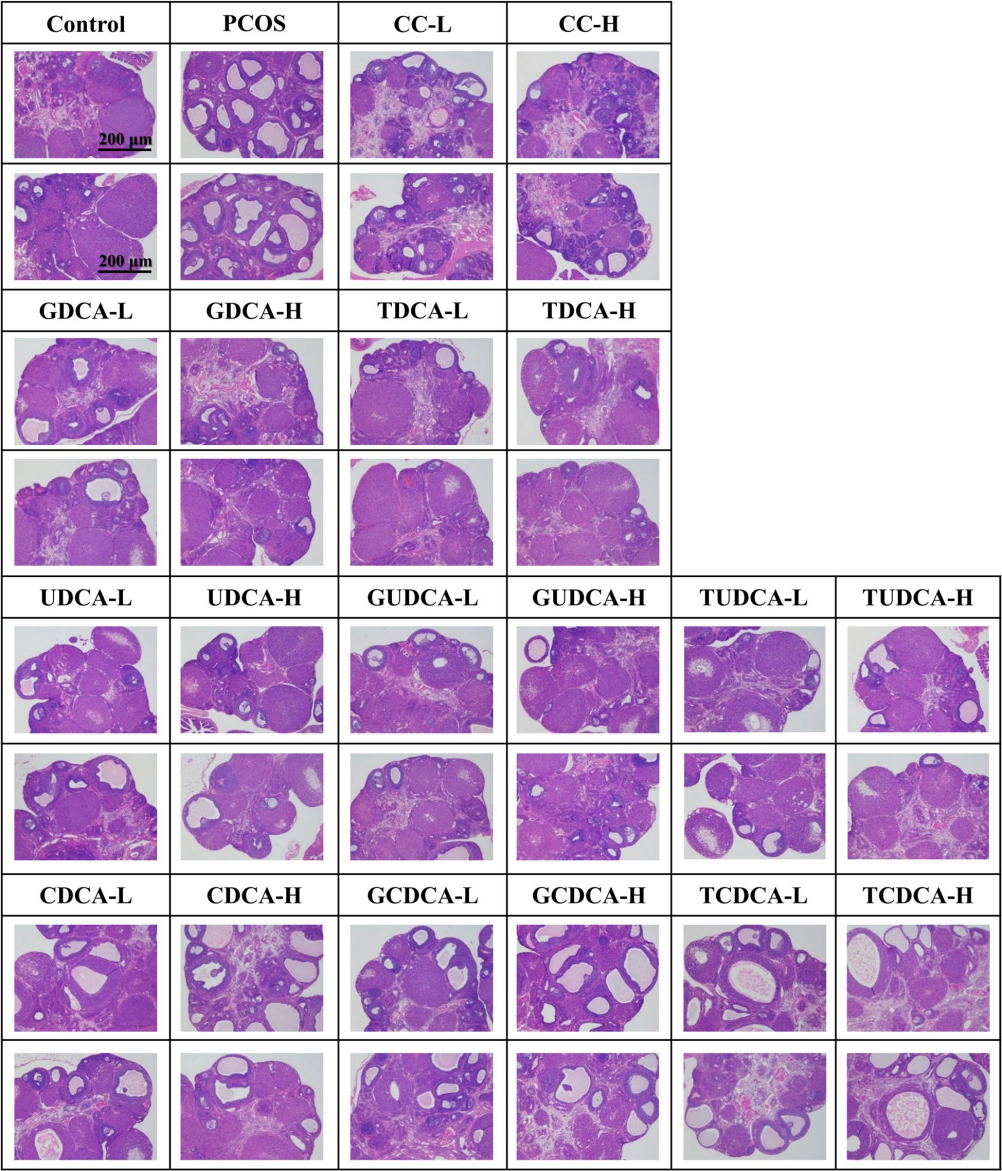
Comparative histopathological analysis of ovarian tissues in the control, PCOS and treatment groups. This figure presents a series of histopathological images of ovarian tissue from different experimental groups, with two representative samples listed for each group. Each image is stained with hematoxylin and eosin (H&E) to visualize the ovarian structure and cellular details. All images are captured at 40 × magnification, with scale bars representing 200 μm. The histopathological changes, such as follicular development and cyst formation, can be compared across the different treatment groups to assess the effects of each bile acid on PCOS pathology

Compared with those in **group 2**, all bile acid-treated groups (**group 5–20)** presented a significant reduction in the number of ovarian cystic follicles (Fig. 4A, Supplementary Table 2). The atretic follicle counts were significantly greater in **group 2** than in **group 1** and several bile acid-treated groups (**group 6**, **8**, **9**, **11** and **14**) (Fig. 4B, Supplementary Table 2). However, no significant difference was observed in the proportion of atretic follicles between these treatment groups (**group 3–14**) and **group 2** (Fig. 4F, Supplementary Table 2). Notably, the CDCA (**group 15** and **16**, *P* < 0.01 and *P* < 0.001), GCDCA (**group 17** and **18**, *P* < 0.01 and *P* < 0.01), and TCDCA (**group 19** and **20**, *P* < 0.01 and *P* < 0.01) groups presented a significantly greater proportion of atretic follicles compared to **group 2** (Fig. 4F, Supplementary Table 2). The average number of antral follicle in the CC (**group 3** and** 4**, *P* < 0.001 and *P* < 0.01), UDCA (**group 7** and **8**, *P* < 0.05 and* P* < 0.05), TUDCA (**group 9** and **10**, *P* < 0.05 and *P* < 0.05) and GUDCA (**group 14**, *P* < 0.01) groups were significantly greater than those in **group 2** (Fig. 4C, Supplementary Table 2). Compared with those in **group 2**, the proportions of antral follicles in the CDCA (**group 15** and **16**), GCDCA (**group 17**), TCDCA (**group 19**), and GUDCA (**group 13**) groups were not significantly different, whereas those in the other treatment groups were significantly greater (Fig. 4G, Supplementary Table 2). In terms of the average corpus luteum count, there was no difference among the CDCA groups (**group 15** and **16**), GCDCA groups (**group 17** and **18**), TCDCA group (**group 20**) and **group 2**, whereas the other treatment groups presented significant increases compared with **group 2** (Fig. 4D, Supplementary Table 2). Additionally, the proportion of the corpus luteum was not significantly different among the CDCA group (**group 16**), GCDCA group (**group 18**), and **group 2**, but it was significantly greater in the other treatment groups than in **group 2** (Fig. 4H, Supplementary Table 2). Furthermore, the GDCA (**group 5** and **6**), UDCA (**group 7** and **8**), TUDCA (**group 9** and **10**), TDCA (**group 11** and **12**), and GUDCA (**group 13** and **14**) groups presented ordered granulosa cells with complete shapes and radiating oocytes and corona radiata in mature follicles (Fig. 5). After treatment (GDCA, UDCA, TUDCA, TDCA, and GUDCA), the ovarian sections presented more corpus luteum and antral follicles (mature follicles) and fewer cystic and atretic follicles (Fig. 5, Supplementary Table 2), indicating that the pathological state of the ovaries was ameliorated. However, treating rats with the other 3 bile acids, CDCA, GCDCA, and TCDCA, did not significantly alter LETZ-induced ovarian pathology.

### Effects of eight bile acids on hormone levels

**Fig. 6:**
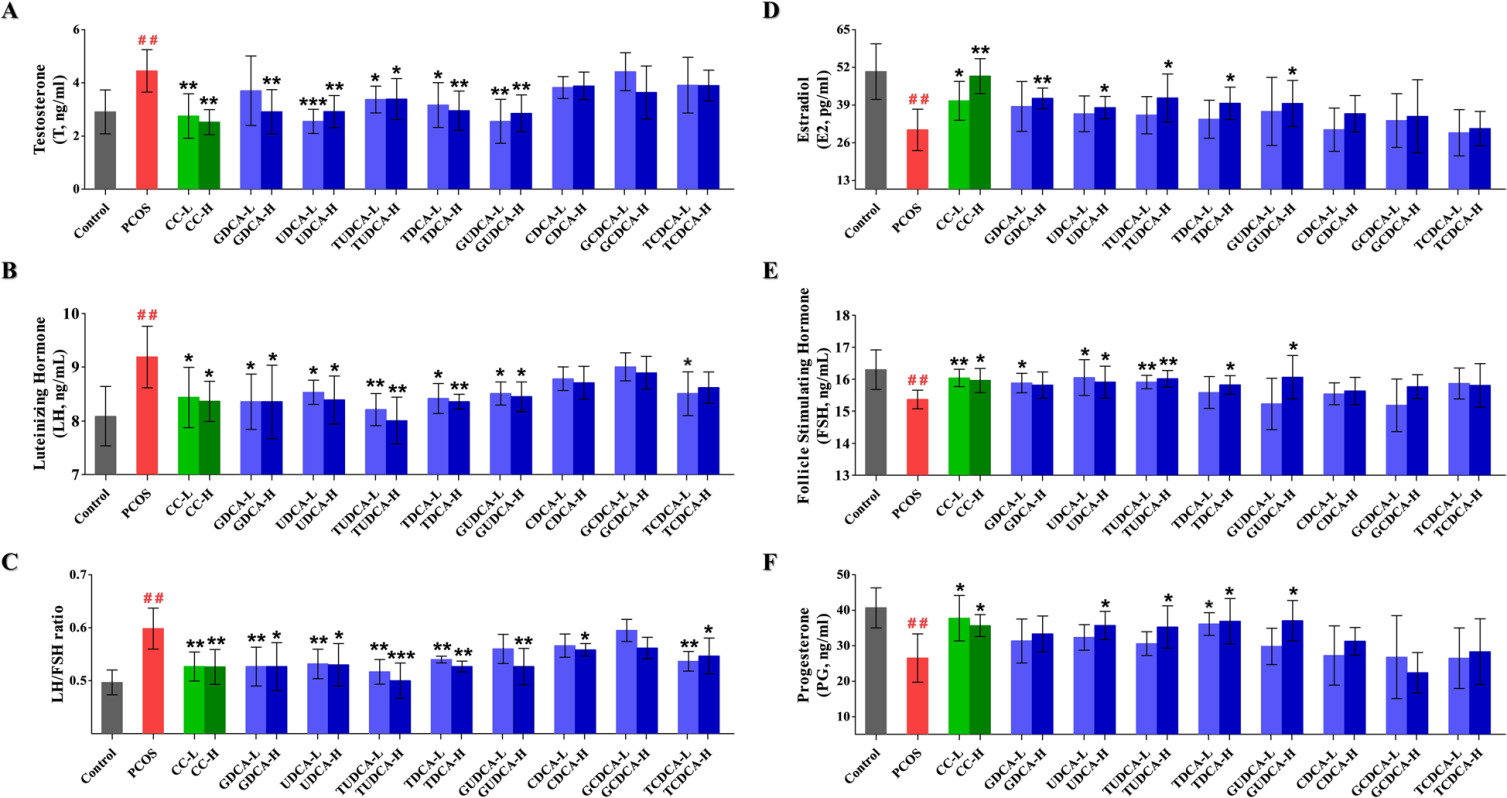
Effects of different bile acid treatments on hormonal profiles in rats with PCOS. This figure presents the hormonal profiles of various groups, including the control, PCOS, and different bile acid treatment groups, at low and high doses. The six subfigures (A-F) depict different hormonal measurements. **A** Shows the concentration of testosterone (T, ng/mL); **B** Illustrates the concentration of luteinizing hormone (LH, ng/mL) across groups; **C** Displays the ratio of luteinizing hormone to follicle-stimulating hormone (LH/FSH); **D** Shows the concentration of estradiol (E2, pg/mL); **E** Illustrates the concentration of follicle-stimulating hormone (FSH, ng/mL); **F** Displays the concentration of progesterone (P4, ng/mL). All data are presented as the mean ± standard deviation (SD), and statistical significance is denoted by asterisks (*) and hashes (^#^). Hashes (^#^): Indicate significant differences between the PCOS group and the control group (^#^, *P* < 0.05; ^##^, *P* < 0.01; ^###^, *P* < 0.001); Asterisks (*): Indicate significant differences between treatment groups and the PCOS group (*, *P* < 0.05; **,* P* < 0.01; ***, *P* < 0.001)

To evaluate the effects of bile acids on hormone imbalances in PCOS rats, an enzyme-linked immunosorbent assay (ELISA) was conducted to measure the levels of T, LH, E2, FSH, and P4 in the plasma of rats from all groups. The results revealed that the plasma levels of T, LH, and the LH/FSH ratio were significantly increased (*P* < 0.01), whereas the levels of E2, FSH, and P4 were significantly decreased (*P* < 0.01) in **group 2** compared with **group 1** (Fig. 6, Supplementary Table 3), demonstrating the characteristic hormone imbalance commonly observed in PCOS individuals. In comparison with **group 2**, the rats treated with CC (**group 3** and **4**) exhibited significant decreases in the plasma levels of T, LH, and LH/FSH ratios along with significant increases in the plasma levels of E2, FSH, and P4 (Fig. 6, Supplementary Table 3), suggesting a near normalization of the hormonal profile. Similarly, treatment groups with GDCA, UDCA, TUDCA, TDCA, and GUDCA exhibited significantly downregulated the levels of T, LH, and LH/FSH ratios (Fig. 6A–C, Supplementary Table 3) compared with **group 2**. Meanwhile, UDCA, TUDCA, TDCA, and GUDCA significantly upregulated the levels of E2, FSH and P4 (Fig. 6D–F, Supplementary Table 3). GDCA notably increased the levels of E2 and FSH (Fig. 6D, E, Supplementary Table 3). However, the other three bile acids (CDCA, GCDCA and TCDCA) did not alter the levels of these hormones in PCOS rats.

### Effects of eight bile acids on chemerin isoform levels

**Fig. 7:**
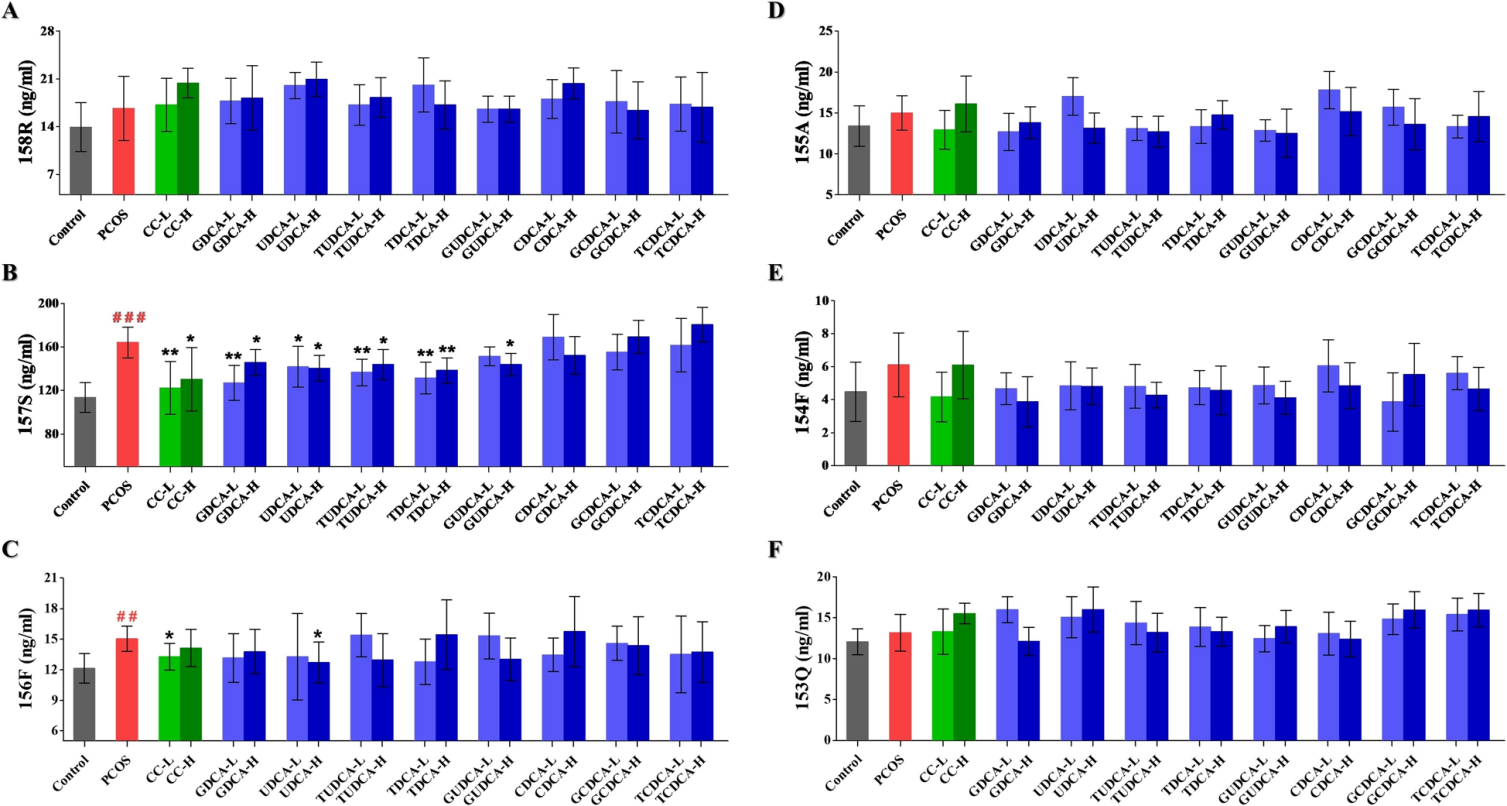
Comparative analysis of chemerin isoform levels in the rats in each group (*n* = 6 rats/group). This figure presents the chemerin isoform levels of various groups, including control, PCOS, and different bile acid treatment groups, at low and high doses. **A** Chemerin-158R (ng/mL); **B** Chemerin-157S (ng/mL); **C** Chemerin-156F (ng/mL); **D** Chemerin-155A (ng/mL); **E** Chemerin-154F (ng/mL); **F** Chemerin-153Q (ng/mL). All data are presented as the mean ± standard deviation (SD), and statistical significance is denoted by asterisks (*) and hashes (^#^). Hashes (^#^): Indicate significant differences between the PCOS group and the control group (^#^, *P* < 0.05; ^##^, *P* < 0.01; ^###^, *P* < 0.001); Asterisks (*): Indicate significant differences between treatment groups and the PCOS group (*, *P* < 0.05; **,* P* < 0.01; ***, *P* < 0.001)

A series of unique peptides of rat chemerin isoforms were synthesized as reference standards for LC–MS analysis. The sequences started at 147I, which is consistent with the tryptic digestion, and ended in the range of 153Q to 158R. Using the corresponding SIS peptides and the three analyte-specific MRM criteria (precursor ion m/z, fragment ion m/z, collision energy and retention time), a method with very high specificity for the analyte was established. The optimized selections were validated with Skyline software (Seattle Proteome Center). The most abundant ion pair was selected as the quantifier, while all others that maintained the same ratio in both the buffer and matrix were selected as qualifiers (Supplementary Table 4). Compared with the synthesized reference standard peptides, all six main isoforms, namely, chemerin-153Q, chemerin-154F, chemerin-155A, chemerin-156F, chemerin-157S, and chemerin-158R, can be characterized and quantified simultaneously. The levels of chemerin-157S and chemerin-156F were significantly elevated in the serum of PCOS rats (**group 2**) compared with those in the controls (Fig. 7B and C, Supplementary Table 5). However, there were no differences in the levels of chemerin-153Q, chemerin-154F, chemerin-155A, and chemerin-158R among the groups (Fig. 7A and D, E, Supplementary Table 5). Furthermore, treatment with CC significantly decreased the levels of chemerin-157S and chemerin-156F compared with those in **group 2** (Fig. 7B and C, Supplementary Table 5). Additionally, the serum levels of chemerin-156F were significantly reduced following treatment with high-dose UDCA, whereas no significant differences were observed among the groups treated with other bile acids (Fig. 7C, Supplementary Table 5). The serum levels of chemerin-157S were significantly lower in the 5 bile acids (GDCA, UDCA, TUDCA, TDCA, and GUDCA) treated groups than in **group 2** (Fig. 7B, Supplementary Table 5).

### Correlation analysis of chemerin-157S with other indicators

**Fig. 8:**
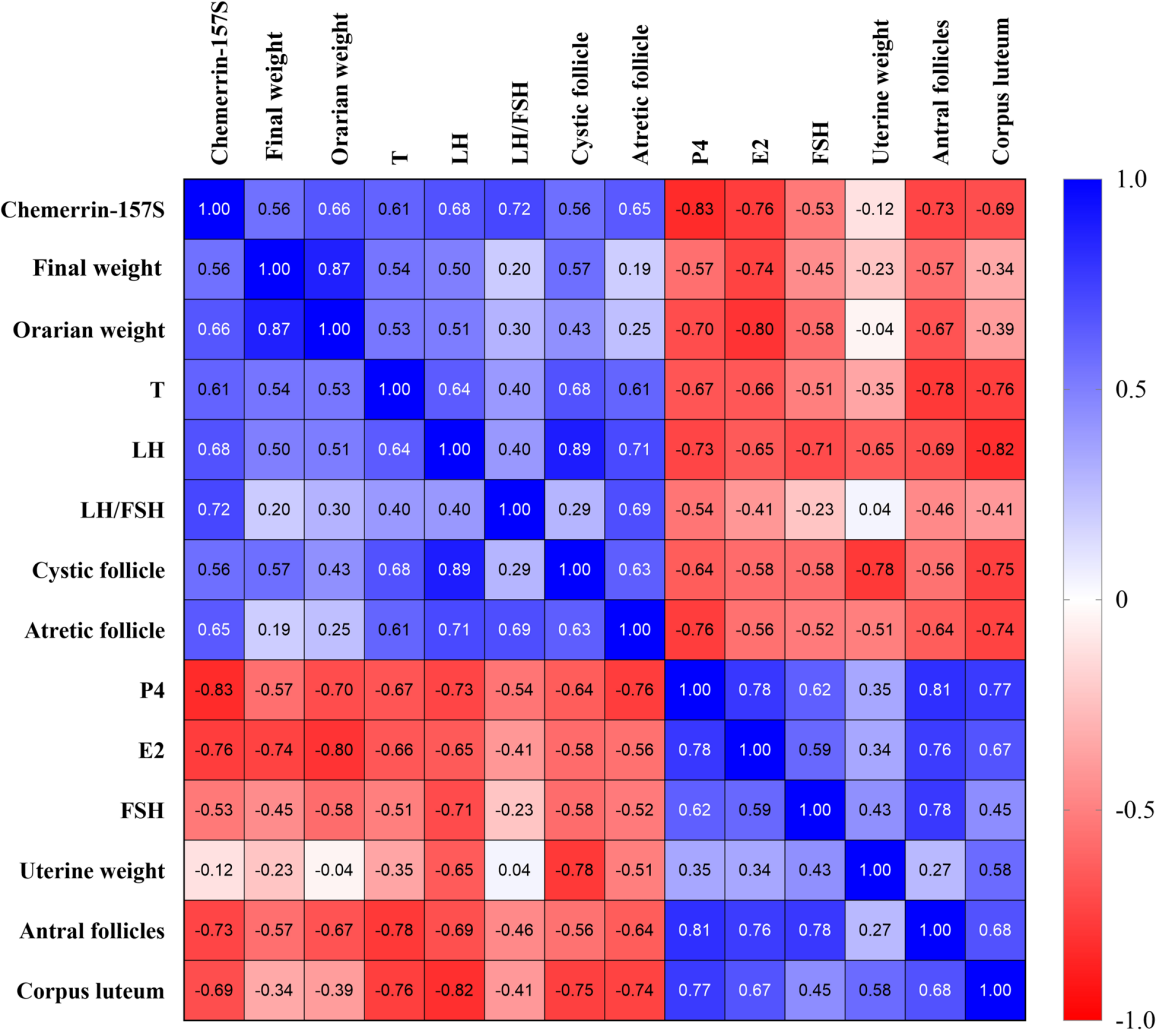
Correlation heatmaps of various biological parameters associated with PCOS. This heatmap illustrates the Pearson correlation coefficients among various biological parameters in this study. Each cell represents the correlation coefficient (r) between one parameter and the corresponding parameter. The color gradient ranges from red (negative correlation) to blue (positive correlation), with the intensity indicating the strength of the correlation

A correlation analysis explored the relationships between Chemerin-157S and a suite of indicators across various groups, including the PCOS, control and treatment groups. This analysis was visually represented through a correlation heatmap, as illustrated in Fig. 8. The heatmap employed a color-coded system to convey the strength and direction of the correlations, providing a clear and intuitive understanding of the statistical interdependencies within the dataset. Chemerin-157S was significantly correlated with several indicators. First, a strong positive correlation was observed between chemerin-157S and the LH/FSH ratio (r = 0.7191, *p* = 0.0004), suggesting that increased chemerin-157S levels could be associated with a higher LH/FSH ratio. Furthermore, chemerin-157S had a moderate positive correlation with LH levels (r = 0.6775, *p* = 0.0010), ovarian weight (r = 0.6625, *p* = 0.0015), atretic follicle counts (r = 0.6493, *p* = 0.0019), and T levels (r = 0.6106, *p* = 0.0042), implying that these indicators might be influenced by chemerin-157S levels. Conversely, a strong negative correlation was noted with P4 (r = − 0.8322, *p* < 0.0001), E2 (r = − 0.7620, *p* < 0.0001), and antral follicles (r = − 0.7297, *p* = 0.0003), indicating that higher chemerin-157S levels might be linked to lower P4 levels, E2 levels, and fewer antral follicles. Finally, a moderate negative correlation was observed between chemerin-157S and the corpus luteum (r = − 0.6945, *p* = 0.0007), suggesting a potential inverse relationship between chemerin-157S and corpus luteum development.

### Exploring potential mechanisms via network pharmacology prediction and molecular docking strategies

**Fig. 9:**
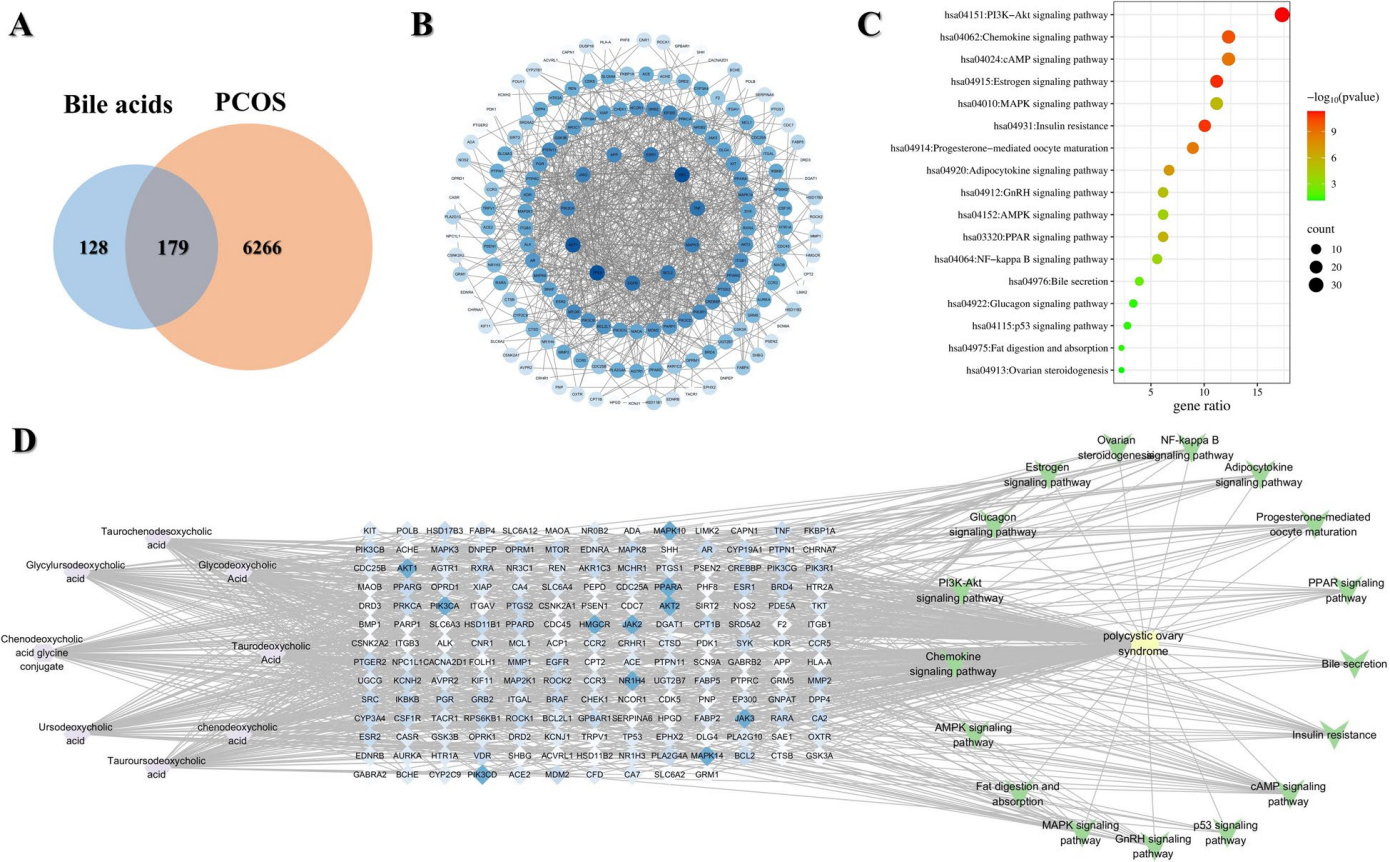
Network pharmacology analysis of bile acids for the treatment of PCOS. **A** Venn diagram of bile acid targets and PCOS targets: The blue circle represents the targets of bile acids, and the orange circle represents the targets of PCOS, and the intersection of the two circles represents the targets of bile acids for PCOS. **B** PPI network of related targets: The nodes represent potential therapeutic targets of bile acids against PCOS. The larger the node is, the higher the target degree and the greater the number of connections to other nodes. PPI = protein–protein interaction. **C** The top 17 KEGG-enriched pathways: This bubble chart presents various signalling pathways involved in PCOS. Each bubble represents a signalling pathway, with the size of the bubble corresponding to the gene ratio (number of involved genes over total pathway genes) and the color indicating the significance level based on the − log_10_ (P-value). **D** Integrated molecular interaction map: This comprehensive map integrates data from the previous panels (**A**–**C**), showing the intricate network of interactions between bile acids, targets, and signalling pathways associated with PCOS. It provides a holistic view of the underlying molecular mechanisms of this disease. The targets section links bile acids and signalling pathways related to PCOS, where each blue diamond node represents a gene or protein. The deeper the color of the node, the greater its degree value, indicating that the gene or protein is of greater significance

The potential targets of each bile acid were predicted using the SwissTargetPrediction database. Subsequently, any duplicate targets were removed, and the remaining targets were merged. This process yielded a total of 307 unique targets related to these eight bile acids. Moreover, a comprehensive search of the GeneCards database identified 6445 targets associated with PCOS. When these targets were imported into the online bioinformatics drawing tool platform, 179 common targets were identified and considered potential therapeutic targets for the treatment of PCOS (Fig. 9A). To further explore the relationships among these 179 potential therapeutic targets, the STRING database was utilized to construct a PPI network, which visually represents the complex interactions among these targets (Fig. 9B). Additionally, KEGG pathway enrichment analysis was performed to identify the metabolic pathways of the potential therapeutic targets for PCOS treatment. After filtering and ranking, the top 17 pathways were depicted in a bubble chart (Fig. 9C). Notable pathways include the PI3K-Akt signalling pathway, chemokine signalling pathway, estrogen signalling pathway, cAMP signalling pathway, MAPK signalling pathway, and adipocytokine signalling pathway. The bile acid-disease target-signalling pathway depicted in Fig. 9D, clearly revealed that bile acids play a central role in alleviating the symptoms and progression of PCOS. The key potential therapeutic targets highlighted in this pathway include NR1H4, PIK3CA, PIK3CD, AKT1, AKT2, JAK2, JAK3, MAPK10, MAPK14, and PPARA.

**Fig. 10:**
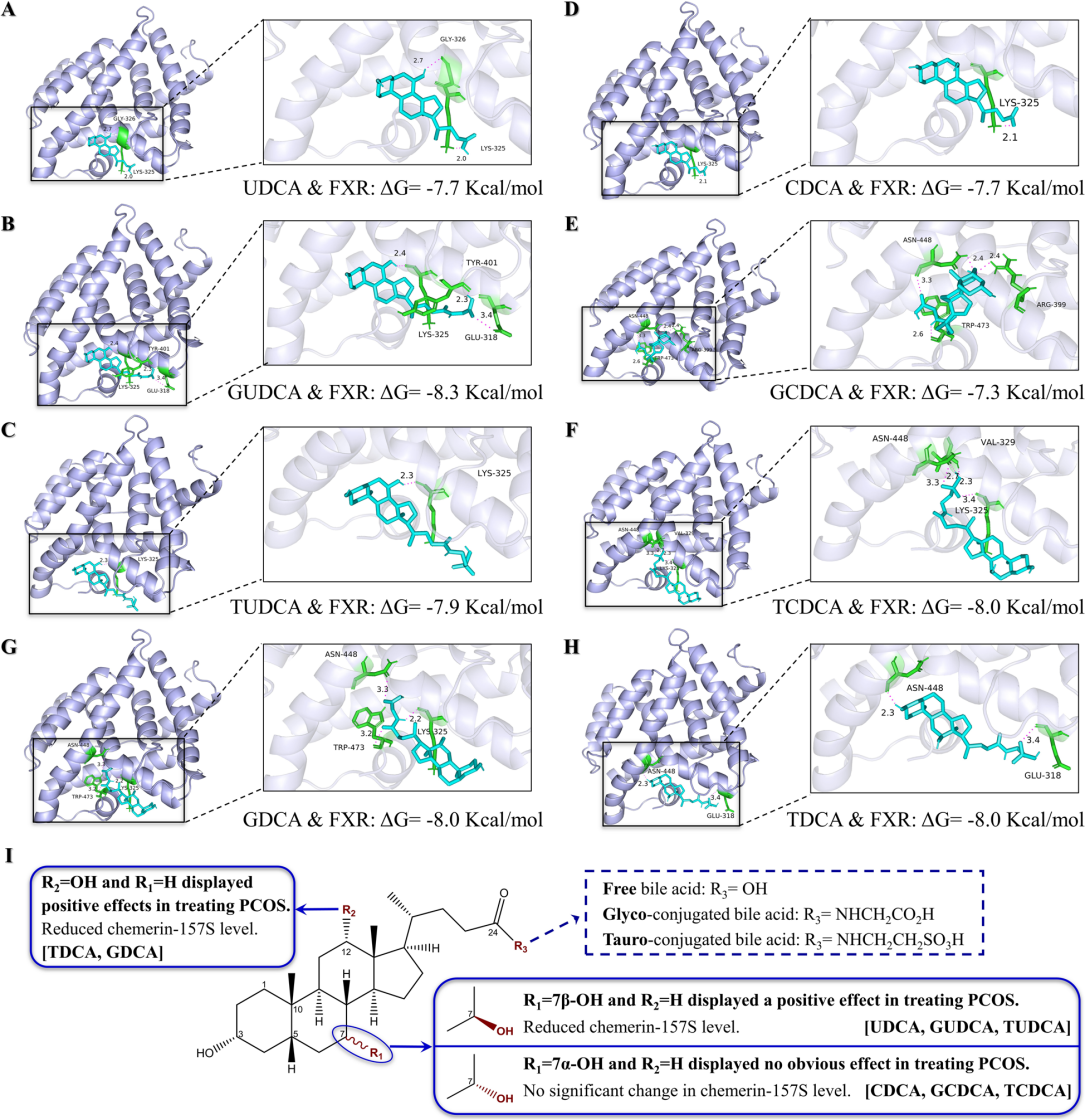
Molecular docking of bile acids with FXR and the structure–activity relationship of bile acids in the treatment of PCOS. **A**–**H** Molecular docking simulations illustrating the binding of various bile acids to FXR, along with their corresponding binding free energies (ΔG) in Kcal/mol. Each figure shows the 3D structure of FXR with the docked bile acid in the binding site, highlighting key interactions. **A** UDCA with FXR: ΔG = − 7.7 kcal/mol; **B** GUDCA with FXR: ΔG = -8.3 kcal/mol; **C** TUDCA with FXR: ΔG = − 7.9 kcal/mol; **D** CDCA with FXR: ΔG = -7.7 kcal/mol; **E** GCDDCA with FXR: ΔG = − 7.3 kcal/mol; **F** TCDDCA with FXR: ΔG = -8.0 kcal/mol; **G** GDCA with FXR: ΔG = − 8.0 kcal/mol; **H** TDCA with FXR: ΔG = -8.0 kcal/mol. **I** The figure illustrates how different substituents (R_1_, R_2_, and R_3_) on the bile acid structure influence their therapeutic effects on PCOS and their impact on chemerin-157S levels. Bile acids with R₂ = OH and R₁ = H (e.g., TDCA, GDCA) exhibit positive therapeutic effects on PCOS and decrease chemerin-157S levels. Those with R₁ = 7*β*-OH and R₂ = H (e.g., UDCA, GUDCA, TUDCA) also have positive effects, whereas bile acids with R₁ = 7*α*-OH and R₂ = H (e.g., CDCA, GCDDCA, TCDDCA) do not show significant effects on PCOS or chemerin-157S levels. Bile acids can be conjugated with either glycine or taurine, usually at the carbon-24 position (R_3_ = NHCH_2_CO_2_H or NHCH_2_CH_2_SO_3_H)

To validate the binding affinity between bile acids and the key target protein FXR (NR1H4), molecular docking was conducted via AutoDockTools-1.5.6. The evaluation parameter for the docking results was ΔG (measured in kcal/mol), which represents the free binding energy between the receptor protein and ligand molecules. A ΔG value of less than − 7.0 kcal/mol indicated that the receptor and ligand could bind to each other under natural conditions [36]. The docking scenarios and energies of bile acids with FXR were illustrated in Fig. 10A‒H. Specifically, CDCA formed hydrogen bonds with the LYS-325 amino acid residue on FXR. GCDCA established hydrogen bonds with the ASN-448, TRP-473, and ARG-399 residues on FXR. TCDCA interacted via hydrogen bonds with the ASN-448, VAL-329, and LYS-325 residues on FXR. UDCA bond through hydrogen bonds to the GLY-326 and LYS-325 residues on FXR. GUDCA connected with the TYR-401, GLU-318, and LYS-325 residues on FXR via hydrogen bonds. TUDCA formed a hydrogen bond with the LYS-325 residue on FXR. TDCA engaged in hydrogen bonding with the ASN-448 and GLU-318 residues on FXR. Finally, GDCA created hydrogen bonds with ASN-448, TRP-473, and LYS-325 residues on FXR.

## Discussion

In this study, LETZ-induced PCOS rats presented significant weight gain, increased ovarian weight, and hormonal imbalances, as well as ovarian pathological changes that align with the clinical manifestations of PCOS. Moreover, we observed a significantly elevated level of chemerin-157S in PCOS rats, which corroborates our previous findings in PCOS patients [31]. Accumulating evidence indicates that the chemerin system plays a role in normal reproductive processes and contributes to the pathological mechanisms of various reproductive system diseases, such as PCOS, preeclampsia, and gynecological cancers [7, 37]. Exogenous recombinant chemerin protein has been reported to induce granulosa cell apoptosis and inhibit follicle growth in vitro [38]. Similarly, locally enhanced active fragments of chemerin have been shown to negatively affect follicular development and oocyte maturation [7]. In the present study, correlation analysis revealed a robust positive relationship between chemerin-157S level and the LH/FSH ratio or LH level (Fig. 8). Elevated level of LH could exacerbate the production of androgens in the ovaries, whereas a subsequent decrease in the level of FSH can impair the normal progression of follicular development [39]. These results suggested that elevated level of chemerin-157S may aggravate ovarian deterioration in PCOS. Furthermore, we observed a significant negative correlation between increased level of chemerin-157S and decreased levels of E2 and P4 (Fig. 8). E2, as a primary estrogen, is crucial for the development and maturation of follicles [40], while P4 regulates essential physiological processes, including the menstrual cycle, implantation, and pregnancy maintenance [41]. Given the critical roles of E2 and P4 in ovarian function, the negative impact of chemerin-157S on these hormones could be a significant contributing factor to the pathophysiology of PCOS. Additionally, histopathological analysis of ovaries from letrozole-induced PCOS rats showed significant alterations in follicular development and ovarian structure, characterized by a higher number of cystic and atretic follicles, and a lower number of antral follicles and corpus luteum. (Fig. 4). Importantly, these abnormal follicular changes in PCOS rats were accompanied by a significant increase in the level of chemerin-157S, further underscoring the role of chemerin-157S in controlling follicular development. In brief, chemerin-157S exerts a substantial influence on hormonal balance and ovarian health, suggesting that intervention of chemerin-157S level may represent a new therapeutic avenue for treating PCOS. Interestingly, administration of GDCA, UDCA, TUDCA, GUDCA, and TDCA to PCOS rats significantly improved the severity of disease, accompanied by restored chemerin-157S levels.

Based on network pharmacology analysis, we constructed a bile acids-disease target-signalling pathways network. Within this network, several highly significant gene-enriched signalling pathways closely associated with chemerin (RARRES2), including the PI3K-Akt signalling pathway, chemokine signalling pathway, cAMP signalling pathway, and MAPK signalling pathway, were identified. However, RARRES2 has not been identified as a direct target; instead, FXR (NR1H4) has emerged as a key target of interest. FXR, a bile acid nuclear receptor, plays a pivotal role in regulating bile acid metabolism. FXR typically forms a heterodimer with the retinoid X receptor (RXR), which can then bind to specific DNA sequences (e.g., FXR response elements, FXRE) in the promoter regions of target genes to regulate gene expression primarily in the liver, intestine, and kidney [42, 43]. Notably, excessive FXR activation inhibits bile acid production by upregulating transrepressor small heterodimer partner 1 (SHP-1), which subsequently represses CYP7A1, a key enzyme in bile acid synthesis [44]. These findings underscore critical role of FXR in maintaining bile acid homeostasis [44]. The FXR agonist GW4064 has been demonstrated to increase chemerin levels in HepG2 cells and primary hepatocytes [45]. This effect is abrogated upon FXR knockout, indicating that FXR regulates chemerin expression [45]. FXRE is located in the -258/-90 base pair region of the RARRES2 (chemerin gene) promoter [45], providing a molecular basis for the direct transcriptional regulation of the chemerin gene by FXR. Therefore, this FXR-chemerin interaction may play a significant role in the pathophysiology of PCOS. Future research will further explore the mechanistic links between FXR activation, chemerin expression, and PCOS-related metabolic disturbances.

Bile acids are natural ligands for FXR and play a critical role in modulating metabolism and cellular functions via their interactions with FXR [46]. Molecular docking studies revealed that there were no significant differences in the binding energies between FXR and the eight bile acids tested, despite their distinct therapeutic effects on PCOS. This observation suggests that while all bile acids can bind to FXR, the differences in their functional outcomes are attributed primarily to inherent structural differences. CDCA is recognized as a natural agonist of human FXR, effectively activating FXR and significantly impacting bile acid synthesis and lipid metabolism [47]. TCDCA has been shown to activate the FXR-SHP-FOXO1 signalling pathway, which plays a role in preventing metabolic disorders in mice [48]. GCDCA may induce hepcidin expression and reduce serum iron levels by activating FXR-related SMAD signalling, thereby affecting iron-related diseases [49]. In this study, these three FXR agonists exhibited minimal changes in most PCOS indicators and chemerin-157S levels. These bile acids share a common steroid core structure containing a 7*α*-hydroxy group, and the R_3_ group, which can be replaced by an amino acid, is distinguished from conjugated bile acids (Fig. 10I). It is thus speculated that the 7*α*-hydroxy group may be a crucial structural feature for bile acids to exert agonistic effects. Fujino T., et al. (2004) investigated the effects of CDCA and UDCA, along with their structurally modified analogs on FXR via a cell-based luciferase assay and a coactivator recruitment assay using surface plasmon resonance (SPR) [50]. Their results revealed that CDCA effectively activated FXR, whereas UDCA did not, highlighting the importance of the stereochemistry of the 7*α*-hydroxyl group for FXR activation—a conclusion that is consistent with our findings. UDCA and TUDCA are well-established FXR antagonists used clinically to treat chronic cholestatic liver diseases [51, 52], while GUDCA has also been confirmed as an FXR antagonist, improving metabolic outcomes in obese mice [53]. The main structural difference from the aforementioned agonists of FXR is that the hydroxyl group at the C-7 position in UDCA, GUDCA, and TUDCA has changed from an* α*-configuration to a *β*-configuration. In our study, UDCA, TUDCA and GUDCA significantly alleviated the severity of disease in PCOS rats while significantly reducing chemerin-157S levels. Molecular docking analysis showed that the 7*β*- hydroxy group of UDCA forms hydrogen bonds with both the GLY-326 and LYS-325 residues of FXR. Similarly, GUDCA and TUDCA bind to FXR via their 7*β*-hydroxy groups, but specifically to LYS-325, not GLY-326. Hence, the 7*β*-hydroxy group may be a key structural feature for bile acids to exert antagonistic effects. Additionally, our animal experiments also showed that GDCA and TDCA could alleviate PCOS severity similarly to FXR antagonists (e.g., UDCA, GUDCA, and TUDCA). It has been reported that TDCA and GDCA act as a partial antagonist by binding to FXR and interfering with the interaction between FXR and its coactivators, such as steroid receptor coactivator-1 (SRC-1) [54]. This result supports our findings, suggesting that these two bile acids may also act as FXR antagonists in treating PCOS. Unlike the above-mentioned bile acids, GDCA and TDCA are characterized by the presence of 12*α*-hydroxyl groups and the absence of 7*α*/*β*-hydroxyl groups, indicating that the 12*α*-hydroxy group may be another factor in their structure-based activity.

## Conclusions

This study investigated the therapeutic potential of eight bile acids in a LETZ-induced PCOS rat model. Our results demonstrated that GDCA, UDCA, TUDCA, TDCA, and GUDCA significantly mitigated PCOS-related indicators, including ovarian pathology, hormonal imbalances, and elevated levels of chemerin-157S. Notably, this study is the first to report the therapeutic effects of TDCA and GUDCA on PCOS, expanding the range of potential bile acid treatments for this condition. This study demonstrated that chemerin-157S is significantly correlated with several PCOS indicators, including the LH/FSH ratio, LH level, T level, ovarian weight, and atretic follicle count, suggesting its potential role in exacerbating ovarian deterioration in PCOS. Network pharmacology analysis identified key signalling pathways, including the PI3K-Akt, chemokine, cAMP, and MAPK signalling pathways, which are closely associated with chemerin and may contribute to the therapeutic effects of bile acids in the treatment of PCOS. Meanwhile, FXR (NR1H4) emerged as a crucial target in the bile acids-disease target-signalling pathway network. Molecular docking studies further elucidated the interactions between bile acids and the FXR, highlighting the importance of specific structural features, such as the 7*β*-hydroxy and 12*α*-hydroxy groups, in modulating FXR activity. Overall, our study highlights the potential therapeutic benefits of bile acids in treating PCOS through targeting chemerin-157S and FXR-related pathways. These findings provide valuable insights into the underlying molecular mechanisms of PCOS and suggest new avenues for therapeutic intervention. Future research should focus on further validating and elucidating the mechanism between FXR activation, chemerin expression, and PCOS-related metabolic disturbances to develop more effective treatments for this condition.

## Supplementary Information

Supplementary Material 1.

## Data Availability

All data for the duration of the study can be found in this article and the appendix.

## Abbreviations

ABC: Ammonium bicarbonate
ACN: Acetonitrile
CC: Clomiphene citrate
CDCA: Chenodeoxycholic acid
CMC-Na: Sodium carboxymethyl cellulose
CMKLR1: Chemokine-like receptor 1
CVD: Cardiovascular disease
E2: Estradiol
ELISA: Enzyme-linked immunosorbent assay
FSH: Follicle stimulating hormone
FXR: Farnesoid X Receptor
FXRE: FXR response element
GCDCA: Sodium glycochenodeoxycholate
GDCA: Glycodeoxycholic acid
GO: Gene Ontology
GUDCA: Glycoursodeoxycholic acid
H&E: Hematoxylin–eosin
HCOOH: Formic acid
KEGG: Kyoto Encyclopedia of Genes and Genomes
LC/dynamic MRM-MS: Liquid chromatography/dynamic multiple reaction monitoring-mass spectrometry
LETZ: Letrozole
LH: Luteinizing hormone
MCC: Maximal Clique Centrality
NAFLD: Non-alcoholic fatty liver disease
OA: Osteoarthritis
P4: Progesterone
PCOS: Polycystic ovary syndrome
PPI: Protein–protein interaction
RA: Rheumatoid arthritis
SHP-1: Small heterodimer partner 1
SMILES: Simplified Molecular Input Line Entry System
SRC-1: Steroid receptor coactivator-1
T: Testosterone
T2DM: Type 2 diabetes
TCDCA: Sodium taurochenodeoxycholate
TCM: Traditional Chinese Medicine
TDCA: Taurodeoxycholic acid
TGR5: Takeda G Protein-Coupled Receptor 5
TSV: Tab-Separated Values
TUDCA: Tauroursodeoxycholic acid
UDCA: Ursodeoxycholic acid
