# Supplementary material for: Pharmacological effects of bile acids on polycystic ovary syndrome via the regulation of chemerin
Source: Chin Med. 2025 Apr 3;20:45. doi: 10.1186/s13020-025-01078-1 (PMC11969753; doi:10.1186/s13020-025-01078-1)
Supplement: Supplementary file 1 — Supplementary Material 1. [file 13020_2025_1078_MOESM1_ESM.docx]

**Supplementary materials**

**Table 1. Changes of body weight, ovarian weight and uterine weight in different groups (*n*=6 rats/group).**

| **Group**  **No.** | **Parameters** | **Day 0**  **body weight (g)** | **Day 21**  **body weight**  **(g)** | **Day 0 to day 21**  **body weight gain**  **(g)** | **Day 35**  **body weight**  **(g)** | **Day 21 to day 35**  **body weight gain**  **(g)** | **Ovarian weight (mg)** | **Uterine weight (mg)** |
| --- | --- | --- | --- | --- | --- | --- | --- | --- |
| **1** | **Control** | 160.30±3.93 | 217.80±6.824 | 57.50±7.23 | 231.7±7.118 | 13.83±5.31 | 114.40±9.63 | 369.80±27.29 |
| **2** | **PCOS** | 162.20±5.04 | 270.50±6.72^###^ | 108.30±3.62 | 326±12.63^###^ | 55.50±6.32 | 209.80±22.94**^###^** | 143.70±20.11**^###^** |
| **3** | **CC-L** | 160.30±4.13 | 262.70±7.74^###^ | 102.30±8.78 | 249.7±7.285*** | -13.00±2.00 | 113.60±25.54*** | 192.40±16.64*** |
| **4** | **CC-H** | 163.20±2.71 | 262.20±4.12^###^ | 99.00±3.69 | 228.2±7.497*** | -33.40±7.40 | 110.50±20.37*** | 272.80±27.51*** |
| **5** | **GDCA-L** | 163.7±4.27 | 259.30±8.07^###^ | 95.67±7.20 | 290.20±6.68*** | 30.83±7.73 | 172.00±12.06** | 268.80±51.61*** |
| **6** | **GDCA-H** | 157.20±3.60 | 254.50±10.75^###^ | 97.33±7.20 | 280.20±7.78*** | 25.67±5.75 | 167.30±23.03** | 383.00±42.06*** |
| **7** | **UDCA-L** | 164.50±4.23 | 267.30±13.52^###^ | 102.80±9.45 | 288.30±12.18*** | 21.00±4.20 | 188.00±33.11 | 295.10±52.33*** |
| **8** | **UDCA-H** | 159.00±3.35 | 253.20±4.92^###^ | 94.17±2.14 | 280.50±7.77*** | 27.33±5.43 | 173.20±26.05* | 319.30±83.22*** |
| **9** | **TUDCA-L** | 159.20±3.49 | 259.80±9.09^###^ | 100.70±5.96 | 281.20±13.60*** | 21.33±6.38 | 196.80±23.44 | 321.70±55.44*** |
| **10** | **TUDCA-H** | 164.80±6.82 | 261.70±10.63^###^ | 96.83±4.36 | 281.80±12.34*** | 20.17±3.92 | 176.10±27.97* | 392.10±39.20*** |
| **11** | **TDCA-L** | 159.30±3.93 | 253.00±10.84^###^ | 93.67±7.76 | 279.50±16.97*** | 26.50±8.69 | 185.60±24.71 | 294.60±70.72*** |
| **12** | **TDCA-H** | 160.00±2.76 | 250.7±11.09^###^ | 90.67±9.29 | 271.80±13.35*** | 21.17±6.80 | 165.10±10.27** | 315.40±54.26*** |
| **13** | **GUDCA-L** | 158.50±5.01 | 256.50±8.55^###^ | 98.00±4.20 | 278.30±6.22*** | 21.83±4.07 | 191.80±26.8 | 299.60±49.44*** |
| **14** | **GUDCA-H** | 162.70±7.89 | 261.50±9.33^###^ | 98.83±4.58 | 287.30±13.26*** | 25.83±7.71 | 184.30±12.80* | 385.10±45.77*** |
| **15** | **CDCA-L** | 162.70±4.41 | 259.30±8.07^###^ | 89.50±5.86 | 276.80±13.92*** | 25.00±8.43 | 189.80±24.22 | 311.00±48.44*** |
| **16** | **CDCA-H** | 158.80±2.64 | 254.50±10.75^###^ | 91.17±11.58 | 276.70±12.96*** | 26.67±7.29 | 187.50±15.17 | 280.70±45.72*** |
| **17** | **GCDCA-L** | 159.70±10.78 | 254.30±7.82^###^ | 94.67±3.08 | 279.30±6.89*** | 25.00±2.83 | 189.40±31.84 | 265.60±44.39*** |
| **18** | **GCDCA-H** | 157.70±4.59 | 258.20±12.02^###^ | 100.50±7.50 | 278.00±16.92*** | 19.83±7.25 | 196.50±19.56 | 293.00±60.52*** |
| **19** | **TCDCA-L** | 165.30±8.07 | 256.00±15.32^###^ | 90.67±7.99 | 280.00±18.88*** | 24.00±4.86 | 183.30±27.21 | 318.40±42.73*** |
| **20** | **TCDCA-H** | 164.00±4.34 | 261.70±12.03^###^ | 97.67±8.07 | 286.50±12.77*** | 24.83±4.31 | 177.50±30.64 | 300.40±70.08*** |

Notes: Values are expressed as mean ± SD.

L: Low dose; H: High dose.

^#^ Significant differences in the PCOS group compared to the control group (^#^, *P* <0.05; ^##,^ *P* <0.01; ^###^, *P* <0.001).

* Significant differences in the treatment group compared to the PCOS group (*, *P* <0.05; **, *P* <0.01; ***, *P* <0.001).

**Table 2. Histologic changes of ovaries in different groups (*n*=6 rats/group).**

| **Group**  **No.** | **Parameters** | **Cystic follicle** | **Cystic follicle**  **(%)** | **Atretic follicle** | **Atretic follicle**  **(%)** | **Antral follicles** | **Antral follicles**  **(%)** | **Corpus luteum** | **Corpus luteum**  **(%)** |
| --- | --- | --- | --- | --- | --- | --- | --- | --- | --- |
| **1** | **Control** | 0.00±0.00 | 0.00±0.00 | 2.50±0.55 | 11.10±1.85 | 8.50±0.84 | 38.00±4.23 | 11.50±2.07 | 50.90±4.01 |
| **2** | **PCOS** | 19.60±4.84^###^ | 67.50±4.75**^###^** | 5.25±1.04^###^ | 18.60±4.27**^##^** | 2.50±1.31^###^ | 8.76±4.73**^###^** | 1.50±1.69^###^ | 5.12±5.93**^###^** |
| **3** | **CC-L** | 5.83±1.47*** | 23.90±5.55*** | 5.33±0.82 | 22.00±3.93 | 7.17±1.72*** | 29.30±6.02*** | 6.17±2.32*** | 24.80±7.40*** |
| **4** | **CC-H** | 3.60±1.52*** | 16.20±7.69*** | 4.40±0.89 | 19.30±3.72 | 6.40±2.70** | 27.50±7.86*** | 8.60±2.70*** | 37.00±7.57*** |
| **5** | **GDCA-L** | 4.00±1.90*** | 18.80±7.85*** | 4.83±0.75 | 23.90±6.82 | 3.50±1.76 | 16.70±8.45* | 8.50±2.66*** | 40.70±9.30*** |
| **6** | **GDCA-H** | 2.00±1.90*** | 10.90±9.66*** | 3.17±0.98** | 16.80±4.68 | 3.50±1.38 | 18.10±6.08** | 10.20±2.93*** | 54.20±13.20*** |
| **7** | **UDCA-L** | 4.00±2.45*** | 19.90±13.10*** | 4.00±1.67 | 18.50±6.38 | 5.83±2.79* | 26.70±9.52*** | 7.83±3.43*** | 35.00±13.40*** |
| **8** | **UDCA-H** | 4.17±1.83*** | 18.10±8.28*** | 3.67±0.82** | 15.90±3.71 | 5.50±2.43* | 23.30±9.12** | 10.00±3.29*** | 42.70±13.30*** |
| **9** | **TUDCA-L** | 2.17±1.60*** | 12.20±8.69*** | 3.00±1.55** | 16.60±8.82 | 5.50±2.95* | 30.40±16.10** | 7.17±2.79*** | 40.70±16.10*** |
| **10** | **TUDCA-H** | 1.67±1.21*** | 7.16±5.21*** | 4.00±1.27 | 17.60±4.55 | 5.17±2.04* | 23.10±8.64** | 11.70±2.07*** | 52.10±8.79*** |
| **11** | **TDCA-L** | 4.00±2.53*** | 19.50±13.30*** | 3.33±1.21** | 15.70±5.87 | 3.83±1.72 | 17.60±5.94** | 10.20±3.71*** | 47.30±16.20*** |
| **12** | **TDCA-H** | 3.33±1.97*** | 14.20±8.14*** | 4.33±1.97 | 18.50±11.90 | 5.33±4.08 | 19.50±12.80* | 12.00±4.56*** | 47.80±14.50*** |
| **13** | **GUDCA-L** | 3.50±2.81*** | 15.80±12.90*** | 4.67±1.86 | 20.50±7.20 | 4.00±2.45 | 17.80±10.80 | 10.30±2.34*** | 45.90±10.20*** |
| **14** | **GUDCA-H** | 2.33±2.58*** | 11.60±13.80*** | 3.00±1.79* | 13.20±5.91 | 6.67±2.58** | 29.60±8.22*** | 10.20±3.54*** | 45.60±14.90*** |
| **15** | **CDCA-L** | 6.40±4.51*** | 33.50±12.30*** | 5.80±0.84 | 35.10±9.25** | 2.20±1.64 | 11.60±7.57 | 3.20±0.837 | 19.80±7.84** |
| **16** | **CDCA-H** | 6.00±3.22*** | 40.70±13.30*** | 4.33±1.21 | 31.50±3.92*** | 1.83±0.75 | 13.60±4.87 | 1.83±1.17 | 14.20±10.70 |
| **17** | **GCDCA-L** | 9.17±3.06*** | 45.10±13.90** | 7.00±2.28 | 34.50±11.30** | 1.00±0.89 | 5.04±4.30 | 3.17±1.17 | 15.40±4.44** |
| **18** | **GCDCA-H** | 9.83±4.83** | 42.10±16.00** | 6.83±1.72 | 31.30±10.10** | 3.33±0.82 | 15.30±4.66* | 2.50±1.05 | 11.40±4.90 |
| **19** | **TCDCA-L** | 5.67±2.34*** | 32.20±7.44*** | 5.83±2.99 | 33.10±12.10** | 2.33±2.50 | 12.20±13.20 | 3.83±1.17* | 22.60±6.06*** |
| **20** | **TCDCA-H** | 6.50±3.39*** | 34.50±9.54*** | 5.83±2.56 | 32.00±8.62** | 3.17±1.72 | 16.50±5.50* | 3.00±1.10 | 17.10±7.04** |

Notes: %: Percentage of ovarian follicles and corpus luteum in different groups, values are expressed as mean ± SD.

L: Low dose; H: High dose.

^#^ Significant differences in the PCOS group compared to the control group (^#^, *P* <0.05; ^##,^ *P* <0.01; ^###^, *P* <0.001).

* Significant differences in the treatment group compared to the PCOS group (*, *P* <0.05; **, *P* <0.01; ***, *P* <0.001).

**Table 3. Comparison of plasma hormone levels in each group of rats (*n*=6 rats/group).**

| **Group No.** | **Parameters** | **T (ng/ml)** | **LH (ng/ml)** | **LH/FSH** | **E2 (pg/ml)** | **FSH (ng/ml)** | **P4 (ng/ml)** |
| --- | --- | --- | --- | --- | --- | --- | --- |
| **1** | **Control** | 2.91±0.83 | 8.09±0.55 | 0.50±0.02 | 50.53±9.59 | 16.29±0.62 | 40.68±5.66 |
| **2** | **PCOS** | 4.45±0.80**^##^** | 9.19±0.57**^##^** | 0.60±0.04**^###^** | 30.46±7.14**^##^** | 15.36±0.29**^##^** | 26.54±6.80**^##^** |
| **3** | **CC-L** | 2.75±0.84** | 8.44±0.56* | 0.53±0.03** | 40.46±6.69* | 16.04±0.27** | 37.76±6.40* |
| **4** | **CC-H** | 2.52±0.47** | 8.36±0.37* | 0.53±0.03** | 48.98±5.99** | 15.96±0.38* | 35.66±3.09* |
| **5** | **GDCA-L** | 3.70±1.31 | 8.36±0.52* | 0.53±0.04** | 38.52±8.62 | 15.88±0.31* | 31.34±6.19 |
| **6** | **GDCA-H** | 2.91±0.83** | 8.35±0.68* | 0.53±0.05* | 41.31±3.57** | 15.81±0.41 | 33.32±5.05 |
| **7** | **UDCA-L** | 2.55±0.45*** | 8.53±0.23* | 0.53±0.03** | 35.99±6.17 | 16.05±0.56* | 32.36±3.60 |
| **8** | **UDCA-H** | 2.92±0.60** | 8.39±0.45* | 0.53±0.04* | 38.12±3.86* | 15.91±0.50* | 35.74±3.96* |
| **9** | **TUDCA-L** | 3.38±0.50* | 8.21±0.30** | 0.52±0.02** | 35.53±6.40 | 15.91±0.21** | 30.58±3.35 |
| **10** | **TUDCA-H** | 3.39±0.77* | 8.01±0.43** | 0.50±0.03*** | 41.43±8.30* | 16.01±0.26** | 35.27±5.97* |
| **11** | **TDCA-L** | 3.17±0.84* | 8.42±0.27* | 0.54±0.01** | 34.12±6.57 | 15.58±0.50 | 36.11±3.17* |
| **12** | **TDCA-H** | 2.95±0.74** | 8.36±0.14** | 0.53±0.01** | 39.60±5.59* | 15.82±0.29* | 36.91±6.41* |
| **13** | **GUDCA-L** | 2.55±0.83** | 8.51±0.21* | 0.56±0.03 | 36.80±11.73 | 15.23±0.80 | 29.81±5.13 |
| **14** | **GUDCA-H** | 2.85±0.69** | 8.45±0.28* | 0.53±0.03** | 39.55±7.98* | 16.06±0.68* | 37.05±5.71* |
| **15** | **CDCA-L** | 3.82±0.41 | 8.78±0.22 | 0.57±0.02 | 30.52±7.44 | 15.54±0.34 | 27.24±8.34 |
| **16** | **CDCA-H** | 3.88±0.52 | 8.71±0.31 | 0.56±0.01* | 36.01±6.27 | 15.63±0.43 | 31.25±3.92 |
| **17** | **GCDCA-L** | 4.42±0.72 | 9.00±0.26 | 0.60±0.02 | 33.66±9.26 | 15.18±0.82 | 26.78±11.72 |
| **18** | **GCDCA-H** | 3.64±0.99 | 8.89±0.31 | 0.56±0.02 | 35.11±12.60 | 15.76±0.37 | 22.38±5.68 |
| **19** | **TCDCA-L** | 3.91±1.05 | 8.51±0.40* | 0.54±0.02** | 29.46±7.98 | 15.86±0.48 | 26.48±8.54 |
| **20** | **TCDCA-H** | 3.90±0.58 | 8.62±0.29 | 0.55±0.03* | 30.91±5.92 | 15.80±0.68 | 28.30±9.26 |

Notes: Values are expressed as mean ± SD.

L: Low dose; H: High dose.

^#^ Significant differences in the PCOS group compared to the control group (^#^, *P* <0.05; ^##,^ *P* <0.01; ^###^, *P* <0.001).

* Significant differences in the treatment group compared to the PCOS group (*, *P* <0.05; **, *P* <0.01; ***, *P* <0.001).

**Table 4. Optimized dynamic MRM parameters for the quantification of rat chemerin isoforms.**

| **Name** | **Target peptide sequence** | **t_R_**  **(min)** | **Dynamic MRM transition** | | **CE**  **(V)** | **Product ion** |
| --- | --- | --- | --- | --- | --- | --- |
|  |  |  | **Q1 (*m/z*)** | **Q3 (*m/z*)** |  |  |
| **153Q-NAT** | **IYFFPGQ** | **9.5** | **436.2211** | **301.1506** | **5** | **P [y3 ^+^]** |
| 153Q-NAT | IYFFPGQ | 9.5 | 436.2211 | 277.1547 | 5 | Y [b2 ^+^] |
| 153Q-NAT | IYFFPGQ | 9.5 | 436.2211 | 147.0764 | 5 | Q [y1 ^+^] |
| **153Q-SIS** | **IYFF (13C9,15N)PGQ** | **9.5** | **441.2347** | **301.1506** | **5** | **P [y3 ^+^]** |
| 153Q-SIS | IYFF (^13^C_9_,^15^N)PGQ | 9.5 | 441.2347 | 277.1547 | 5 | Y [b2 ^+^] |
| 153Q-SIS | IYFF (^13^C_9_,^15^N)PGQ | 9.5 | 441.2347 | 147.0764 | 5 | Q [y1 ^+^] |
| **154F-NAT** | **IYFFPGQF** | **11.8** | **509.7553** | **448.2191** | **5** | **P [y4 ^+^]** |
| 154F-NAT | IYFFPGQF | 11.8 | 509.7553 | 571.2915 | 5 | F [b4 ^+^] |
| 154F-NAT | IYFFPGQF | 11.8 | 509.7553 | 424.2231 | 5 | F [b3 ^+^] |
| **154F-SIS** | **IYFFPGQF (13C9,15N)** | **11.8** | **514.7689** | **458.2463** | **5** | **P [y4 ^+^]** |
| 154F-SIS | IYFFPGQF (^13^C_9_,^15^N) | 11.8 | 514.7689 | 571.2915 | 5 | F [b4 ^+^] |
| 154F-SIS | IYFFPGQF (^13^C_9_,^15^N) | 11.8 | 514.7689 | 424.2231 | 5 | F [b3 ^+^] |
| **155A-NAT** | **IYFFPGQFA** | **11.5** | **545.2738** | **519.2562** | **5** | **P [y5 ^+^]** |
| 155A-NAT | IYFFPGQFA | 11.5 | 545.2738 | 853.4243 | 5 | Q [b7 ^+^] |
| 155A-NAT | IYFFPGQFA | 11.5 | 545.2738 | 424.2231 | 5 | F [b3 ^+^] |
| **155A-SIS** | **IYFFPGQF (13C9,15N) A** | **11.5** | **550.2874** | **529.2834** | **5** | **P [y5 ^+^]** |
| 155A-SIS | IYFFPGQF (^13^C_9_,^15^N) A | 11.5 | 550.2874 | 853.4243 | 5 | Q [b7 ^+^] |
| 155A-SIS | IYFFPGQF (^13^C_9_,^15^N) A | 11.5 | 550.2874 | 424.2231 | 5 | F [b3 ^+^] |
| **156F-NAT** | **IYFFPGQFAF** | **13.5** | **618.8080** | **666.3246** | **8** | **P [y6 ^+^]** |
| 156F-NAT | IYFFPGQFAF | 13.5 | 618.8080 | 571.2915 | 5 | F [b4 ^+^] |
| 156F-NAT | IYFFPGQFAF | 13.5 | 618.8080 | 424.2231 | 10 | F [b3 ^+^] |
| **156F-SIS** | **IYFFPGQF (13C9,15N) AF** | **13.5** | **623.8217** | **676.3518** | **5** | **P [y6 ^+^]** |
| 156F-SIS | IYFFPGQF (13C9,15N) AF | 13.5 | 623.8217 | 571.2915 | 5 | F [b4 ^+^] |
| 156F-SIS | IYFFPGQF (13C9,15N) AF | 13.5 | 623.8217 | 424.2231 | 5 | F [b3 ^+^] |
| **157S-NAT** | **IYFFPGQFAFS** | **12.7** | **662.3241** | **753.3566** | **5** | **P [y7 ^+^]** |
| 157S-NAT | IYFFPGQFAFS | 12.7 | 662.3241 | 571.2915 | 5 | F [b4 ^+^] |
| 157S-NAT | IYFFPGQFAFS | 12.7 | 662.3241 | 424.2231 | 5 | F [b3 ^+^] |
| **157S-SIS** | **IYFFPGQF (13C9,15N) AFS** | **12.7** | **667.3377** | **763.3838** | **5** | **P [y7 ^+^]** |
| 157S-SIS | IYFFPGQF (13C9,15N) AFS | 12.7 | 667.3377 | 571.2915 | 5 | F [b4 ^+^] |
| 157S-SIS | IYFFPGQF (13C9,15N) AFS | 12.7 | 667.3377 | 424.2231 | 5 | F [b3 ^+^] |
| **158R-NAT** | **IYFFPGQFAFSR** | **11.2** | **740.3746** | **909.4577** | **15** | **P [y8 ^+^]** |
| 158R-NAT | IYFFPGQFAFSR | 11.2 | 740.3746 | 528.7667 | 5 | F [y9 ^2+^] |
| 158R-NAT | IYFFPGQFAFSR | 11.2 | 740.3746 | 424.2231 | 5 | F [b3 ^+^] |
| **158R-SIS** | **IYFFPGQF (13C6,15N2) AFSR** | **11.2** | **745.3882** | **919.4850** | **15** | **P [y8 ^+^]** |
| 158R-SIS | IYFFPGQF (13C6,15N2) AFSR | 11.2 | 745.3882 | 533.7803 | 5 | F [y9 ^2+^] |
| 158R-SIS | IYFFPGQF (13C6,15N2) AFSR | 11.2 | 745.3882 | 424.2231 | 5 | F [b3 ^+^] |

Notes: NAT- Natural standard; SIS- Stable isotope labeled standard.

Both SIS and NAT peptides have the same chromatographic characteristics with only their mass difference demonstrated by MS. All corresponding transitions remained the same between SIS and NAT. The first transition of each peptide was selected as quantifier.

**Table 5. Comparison of serum chemerin isoform levels in each group of rats (*n*=6 rats/group).**

| **Group No.** | **Parameters** | **153Q (ng/ml)** | **154F (ng/ml)** | **155A (ng/ml)** | **156F (ng/ml)** | **157S (ng/ml)** | **158R (ng/ml)** |
| --- | --- | --- | --- | --- | --- | --- | --- |
| **1** | **Control** | 12.06±1.59 | 4.48±1.79 | 13.41±2.46 | 12.14±1.44 | 113.40±13.73 | 13.92±3.58 |
| **2** | **PCOS** | 13.17±2.25 | 6.11±1.94 | 15.01±2.10 | 15.03±1.24**^##^** | 164.00±14.14**^###^** | 16.68±4.71 |
| **3** | **CC-L** | 13.30±2.76 | 4.17±1.50 | 12.95±2.36 | 13.27±1.32* | 122.00±24.37** | 17.16±3.90 |
| **4** | **CC-H** | 15.53±1.25 | 6.10±2.05 | 16.1±3.39 | 14.14±1.83 | 130.10±29.23* | 20.40±2.16 |
| **5** | **GDCA-L** | 15.98±1.58 | 4.66±0.96 | 12.69±2.27 | 13.15±2.37 | 126.70±16.16** | 17.75±3.34 |
| **6** | **GDCA-H** | 12.10±1.72 | 3.87±1.53 | 13.82±1.93 | 13.77±2.17 | 145.50±12.01* | 18.19±4.72 |
| **7** | **UDCA-L** | 15.08±2.52 | 4.83±1.46 | 17.01±2.29 | 13.27±4.24 | 141.60±18.83* | 20.02±1.93 |
| **8** | **UDCA-H** | 16.00±2.77 | 4.81±1.12 | 13.15±1.85 | 12.71±2.00* | 140.30±11.82* | 20.93±2.51 |
| **9** | **TUDCA-L** | 14.35±2.63 | 4.80±1.33 | 13.09±1.45 | 15.38±2.12 | 136.50±12.24** | 17.18±2.98 |
| **10** | **TUDCA-H** | 13.19±2.37 | 4.27±0.77 | 12.71±1.90 | 12.94±2.59 | 143.70±13.81* | 18.27±2.88 |
| **11** | **TDCA-L** | 13.86±2.38 | 4.73±1.04 | 13.33±2.06 | 12.77±2.24 | 131.20±14.53** | 20.11±3.97 |
| **12** | **TDCA-H** | 13.31±1.78 | 4.55±1.48 | 14.75±1.74 | 15.45±3.41 | 138.30±11.69** | 17.16±3.56 |
| **13** | **GUDCA-L** | 12.45±1.59 | 4.87±1.12 | 12.87±1.31 | 15.31±2.25 | 151.20±8.54 | 16.56±1.90 |
| **14** | **GUDCA-H** | 13.90±2.00 | 4.11±1.01 | 12.52±2.96 | 13.02±2.09 | 143.70±10.48* | 16.56±1.90 |
| **15** | **CDCA-L** | 13.07±2.62 | 6.05±1.58 | 17.8±2.29 | 13.45±1.64 | 168.80±20.86 | 18.05±2.84 |
| **16** | **CDCA-H** | 12.38±2.17 | 4.85±1.40 | 15.17±2.95 | 15.75±3.46 | 152.10±17.38 | 20.33±2.26 |
| **17** | **GCDCA-L** | 14.81±1.88 | 3.86±1.77 | 15.69±2.19 | 14.60±1.69 | 155.10±16.55 | 17.64±4.58 |
| **18** | **GCDCA-H** | 15.96±2.23 | 5.52±1.89 | 13.62±3.14 | 14.35±2.85 | 169.20±15.21 | 16.37±4.20 |
| **19** | **TCDCA-L** | 15.41±2.00 | 5.60±1.00 | 13.34±1.37 | 13.50±3.76 | 161.50±24.76 | 17.30±3.95 |
| **20** | **TCDCA-H** | 15.93±2.04 | 4.64±1.33 | 14.56±3.06 | 13.74±2.97 | 180.40±15.95 | 16.84±5.12 |

Notes: Values are expressed as mean ± SD.

L: Low dose; H: High dose.

^#^ Significant differences in the PCOS group compared to the control group (^#^, *P* <0.05; ^##,^ *P* <0.01; ^###^, *P* <0.001).

* Significant differences in the treatment group compared to the PCOS group (*, *P* <0.05; **, *P* <0.01; ***, *P* <0.001).
